# Yeast-secreted compounds with antifungal activity—screening, genetic parts, biosynthetic pathways, and regulation

**DOI:** 10.1093/femsyr/foaf068

**Published:** 2025-11-19

**Authors:** Alicia Maciá Valero, Min Lu, Sonja Billerbeck

**Affiliations:** Molecular Microbiology, Groningen Biomolecular Sciences and Biotechnology Institute, University of Groningen, 9747 AG Groningen, the Netherlands; Molecular Microbiology, Groningen Biomolecular Sciences and Biotechnology Institute, University of Groningen, 9747 AG Groningen, the Netherlands; Molecular Microbiology, Groningen Biomolecular Sciences and Biotechnology Institute, University of Groningen, 9747 AG Groningen, the Netherlands; Department of Bioengineering, Imperial College London, South Kensington Campus, London SW7 2AZ, United Kingdom

**Keywords:** microbial antagonism, biocontrol, iron siderophores, volatile organic compounds, biosurfactants, mycocins, hydrolytic enzymes, activity screening

## Abstract

Awareness is rising that antifungal resistance poses a threat to agriculture, food safety, biodiversity, and human health. There is a limited number of antifungals available and resistance to all of them has been reported. The development of novel antifungals is complex, as eukaryotic organisms have very few selective drug targets that distinguish them from the infected plant, human, or animal host. Yeasts produce different compounds with antifungal activity, ranging from small molecules such as iron chelators, biosurfactants, and volatile organic compounds, to proteins like myocins and hydrolytic enzymes. Those could be further developed into new antifungals; however, there is a scarcity of fundamental knowledge on their chemical structure, their mode of action, their biosynthesis, and its regulation. Given the opportunities that yeasts display as industrial hosts and the synthetic biology tools available, a deeper understanding of these molecular aspects could enable a wider range of yet underexplored applications for the producer yeast and their molecules, from biocontrol to food preservation and human health. To facilitate this exploration, we here consolidate current molecular knowledge on these compounds, suggest readily available methodologies to screen for different molecule classes in natural yeast isolates and discuss how they could be further studied and engineered towards their eventual application.

## Teaser

This work consolidates current molecular knowledge on antifungal compounds produced by yeasts, highlights screening methodologies for their discovery, and outlines how deeper understanding and engineering could unlock novel applications in biocontrol, food preservation, and human health.

## Introduction

Fungal infections pose a threat to humans, wildlife, and agriculture (Fisher et al. 2016, 2020). Over 1.6 million people die annually worldwide due to fungal infections (Brown et al. 2012a, b, Stop neglecting fungi 2017). Although superficial infections are most prevalent, genera like *Candida, Aspergillus*, and *Cryptococcus* (Brown et al. 2012a) can cause severe systemic infections especially in immunocompromised individuals (Brown et al. 2012a, Arastehfar et al. 2020, Mei-Sheng Riley 2021). Mortality rates are often higher than 50% (Brown et al. 2012a), resulting in death tolls comparable to tuberculosis and higher than malaria (Stop neglecting fungi 2017).

Further, fungi are a major cause of crop diseases. More than 8000 fungal species infect plants (Agrios 2005), which leads to the loss of one third of food crops per year (Stop neglecting fungi 2017, Almeida et al. 2019). In addition, fungi contribute to the 25% spoilage of food supply due to microbial contamination (Fleet 2007, Hocking 2014, Snyder and Worobo 2018).

Only four classes of antifungals are commercially available for use in medicine and agriculture; the azoles, polyenes, pyrimidine, analogues and echinocandins; and resistance to all four classes has been reported (Yourman and Jeffers 1999, Chandenier et al. 2004, Snelders et al. 2008, Verweij et al. 2009, Sun et al. 2010, Smith et al. 2015, Prigent et al. 2016, Sahal and Bilkay 2018, Mroczynska and Brillowska-Dabrowska 2019, Ostrowsky et al. 2020). Consequently, the WHO recently listed several human pathogens as high priority pathogens (World Health Organization 2022). The azoles are coused in agriculture, where they constitute the major class of antifungals. Due to an increase in the worldwide temperature, together with practices like monocrop culturing and excessive use of azole-based pesticides, azole resistance has increased (Verweij et al. 2020). Consequently, the current arsenal of chemical pesticides is failing to tackle fungal pathogens. In addition, the European Commission demands that the use of pesticides gets halved by 2030, as part of their Green Deal (European Commission 2022). Thus, there is an urgent need for novel products with antifungal activity against crop and human fungal pathogens.

Yeasts naturally produce different compounds with antifungal activity, including iron chelators, biosurfactants, volatile organic compounds (VOCs), and proteins, like mycocins and enzymes (reviewed before in Spadaro and Droby 2016, Freimoser et al. 2019, Zhang et al. 2020). Some of these antifungal yeasts and their associated molecules are already used commercially (Table 1), showcasing their immediate application potential for pre- and postharvest biocontrol, and pointing to their potential in food preservation and human health.

**Table 1.** Commercially available yeast-based products.

Company	Product	Organism	Use	Target organism
SAN Agrow, Austria (https://www.san-agrow.com/)	Blossom Protect	*Aureobasidium pullulans* DSM 14940 and DSM 14941	Bioprotection of postharvest stored pome fruits	*Penicillium* spp., *B. cinerea, Monilinia* spp., and *Neofabraea alba*
	Botector	*A. pullulans* DSM 14940 and DSM 14941	Bioprotection of grape, strawberry, and tomato plants	*B. cinerea*
Lamothe-Abiet, France (https://lamothe-abiet.com/)	Excellence B-Nature	*M. pulcherrima*	Bioprotection of postharvest grapes/apples and prevention of wine and cider spoilage	*Brettanomyces* spp. and other non-*Saccharomyces* spp.
Lallemand, Canada (https://www.lallemand.com/)	Gaïa™	*M. pulcherrima* E491	Bioprotection of postharvest grapes and prevention of wine spoilage	*B. cinerea, Hanseniaspora uvarum*
	Lalcafé BSC™	*S. cerevisiae*	Bioprotection during coffee fermentation	Broad spectrum
	Level2 INITIA™	*M. pulcherrima* E491	Bioprotection of white and rosé wine	Broad spectrum
	Level2 GUARDIA™	*M. pulcherrima* E491	Bioprotection of red wine	Broad spectrum
BioNext, the Netherlands (https://bionext.nl/)	Julietta®	Cell wall derivatives from *S. cerevisiae* LAS02	Bioprotection and inducer of plant defence response in grapevine	*Monilinia* spp. and *B. cinerea*
Koppert, the Netherlands (https://www.koppert.nl/)	Nexy	*Candida oleophila* strain O	Bioprotection of postharvest apples and pears	*B. cinerea* and *Penicillium expansum*
Lesaffre, France (https://www.lesaffre.com/)	Noli (formerly Shemer)	*Metschnikowia fructicola* NRRL Y-27 328	Bioprotection on diverse crops and postharvest fruits	*B. cinerea* and *Monilinia* spp.
	Romeo	Cell wall derivatives from *S. cerevisiae* LAS117	Bioprotection and inducer of plant defence response in grapevine	*Erysiphe necator* and *B. cinerea*
Laffort, France (https://laffort.com/)	Zymaflore® Égide	*M. pulcherrima* and *Torulaspora delbrueckii*	Bioprotection of postharvest grapes and juice prior fermentation	Broad spectrum
	Zymaflore® Khio	*M. pulcherrima*	Bioprotection of postharvest grapes and juice prior fermentation	Broad spectrum

Yeasts are unicellular fungi—although some are dimorphic, switching between unicellular and hyphal growth—of phylogenetically diverse origin and show multiple attractive characteristics for industrial and environmental applications: many yeasts grow fast and can reach high cell densities, many are robust to harsh conditions (pH, temperature, osmotic stress, and pressures), and many have simple, thus affordable cultivation requirements. Moreover, due to their clonal nature, many yeasts can be genetically manipulated.

Reports on yeast isolates with an antifungal phenotype are vast (Buzzini and Martini 2000, Carreiro et al. 2002, Vadkertiová and Sláviková 2007, de Ullivarri et al. 2011, da Cunha et al. 2018, Liu et al. 2018), but the molecular underpinnings for their antifungal activity are often unclear, such as the actual molecules produced, their biosynthesis, the mode of action, and regulation. Even commercially available yeast-based products (Table 1) often lack detailed molecular characterization, as well as studies on the environmental conditions required for compound production and a deeper understanding of pathogen–antagonist interactions. These knowledge gaps hinder both product optimization and consistent performance in practical applications (Droby et al. 1998, Kowalska et al. 2012, Spadaro and Droby 2016). Thus, there is a disparity between the amount of reported yeast isolates with antagonistic activity and those that result in commercial products (Spadaro and Droby 2016). Focused research on the molecular aspects of these antifungal activities and their enhancement could aid developing marketable formulations (Spadaro and Droby 2016).

**Figure 1.**
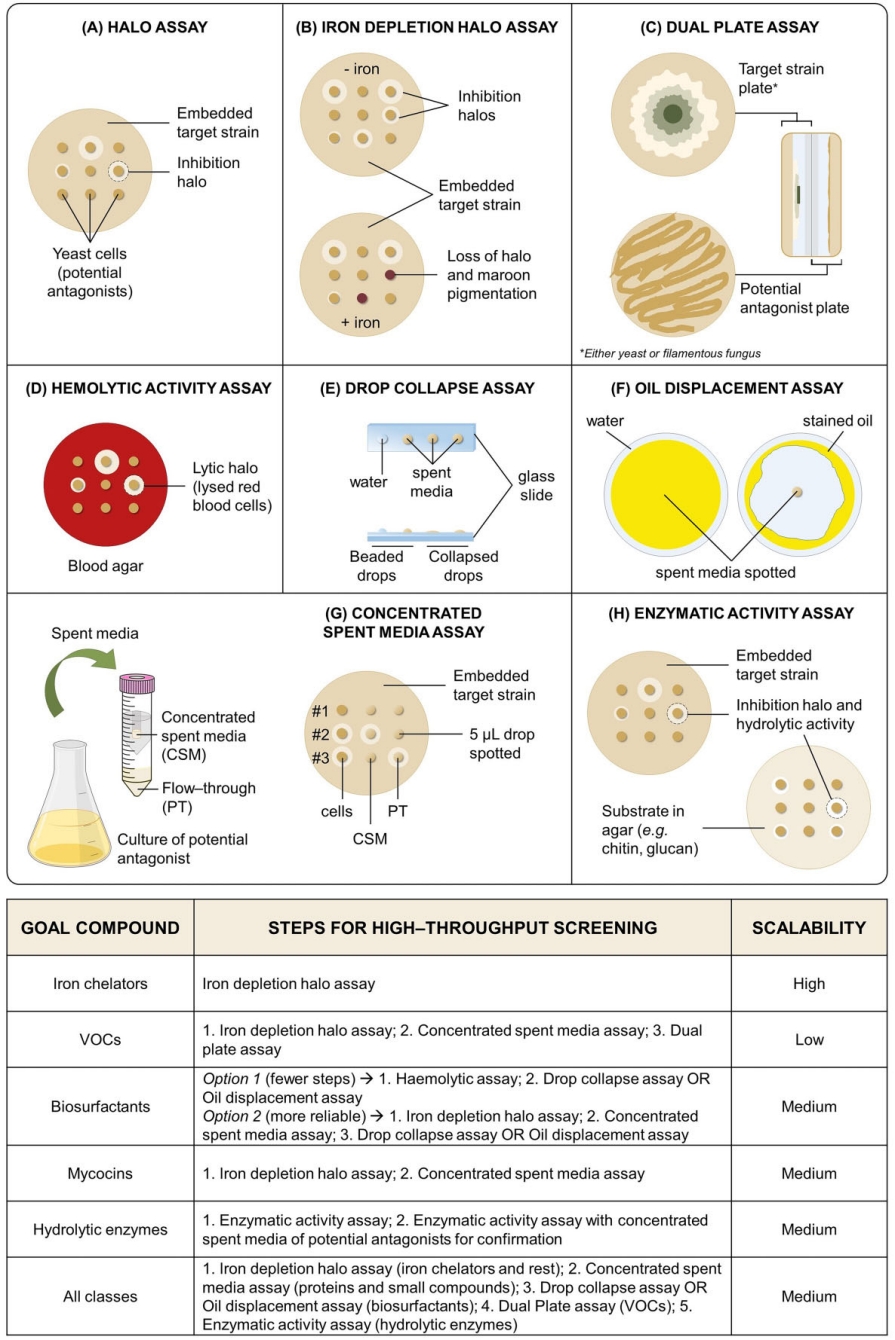
Discriminatory screening assays and workflows for identifying yeast isolates that produce a given compound. (A) Halo assay (Prins and Billerbeck 2025). General assay, where target cells are embedded in agar and yeast cells that are candidates for potential antagonists are spotted on top. The presence of an inhibitory halo indicates the production of a secreted compound with antifungal activity. (B) Iron depletion halo assay (Maciá Valero et al. 2025a). Halo assay in duplicate with and without iron supplementation. This high-throughput capable assay discriminates between isolates that display inhibitory activity based on the production of iron chelators (loss of halo and maroon pigmentation in the presence of iron) and those that produce other compounds responsible for the antifungal phenotype. This assay is limited to the identification of iron chelators that are constitutively produced (e.g. pulcherriminic acid). It is possible—although uncommon—to identify isolates with more than one prominent antifungal phenotype and hence observe cells with maroon pigmentation and no loss of inhibition halo. (C) Dual plate assay (Huang et al. 2011). Two plates facing each other sealed with parafilm where one contains the target strain of interest and the other one contains the potential antagonist. After incubation with no physical contact, any inhibitory phenotype (growth, spore germination, mycelial formation…) indicates the production of VOCs with antifungal activity. The target strain can be either yeast or filamentous fungus. This assay cannot be scalable and should be used as a second step after ruling out other compounds when dealing with large collections of isolates. (D) Haemolytic activity assay (Uyar and Sağlam 2021). Red blood cells are embedded on agar. Cells displaying a surrounding lysis halo indicate the presence of an amphipathic compound able to disrupt cellular membranes. This scalable and inexpensive assays should serve as a preliminary test for the identification of biosurfactants with further confirmation with E or F, since it might lead to false positives. (E) Drop collapse assay (Fernandes et al. 2023). The spent media of potential biosurfactant-producing yeasts is spotted on a glass slide next to water (control). After 1 min, the drops spotted should remain beaded (no change in surface tension) or have collapsed (when containing surfactants) Longer incubations are possible. (F) Oil displacement assay (Fernandes et al. 2023). A Petri dish is covered by water and subsequently with a layer of stained oil (for better readout). Subsequently, the spent media of potential antagonistic yeasts is added in the middle of the plate. After an incubation of 1 min, a ring should appear in the presence of biosurfactants, since those reduce the surface tension of the oil–water interface. Longer incubations are possible. This method has a clearer readout than the drop collapse assay but requires more material. (G) Concentrated spent media assay (Maciá Valero et al. 2025a). The spent media of potential antagonists is concentrated in centrifugal tubes with a molecular size cut-off membrane (it is recommended to use 3 kDa cut-off, the smallest manufactured to date). Cells, concentrated spent media (CSM) and flow-through (PT) are tested. This assay discriminates between yeasts producing proteins (>3 kDa) at least partially responsible for the inhibitory activity—with a halo in cells and CSM (#2) and yeasts producing small compounds—with an inhibitory halo in cells or cells and PT (#3). (H) Enzymatic activity assay (Wu et al. 2018, Gonfa et al. 2023). Cells can be tested in parallel against a target strain and with a substrate of interest on agar (e.g. chitin when screening for chitinase activity). Cells that show a surrounding halo in both plates indicate both production of hydrolytic enzymes and inhibitory activity. This assay is inexpensive and highly scalable, but should be used as a preliminary test; after, an enzymatic activity assay with the CSM instead of cells should be used to confirm that the enzymes secreted are (at least partially) responsible for the antifungal activity observed.

Here, we provide an overview on the known classes of antifungal molecules, which are produced by yeast, focussing on what is known about their biosynthesis, regulation, and antagonistic mode of action, while pointing to important knowledge gaps. Furthermore, we present simple screening assays that allow to elucidate the nature of the secreted molecules and its potential mode of action (Fig. 1)—a first step in finding interesting yeast isolates given a certain research question or application—and we discuss how genetic and metabolic engineering could aid molecular studies and bioproduction.

### Iron chelators produced by yeast: structure and mechanism of inhibition

Iron chelators—often called siderophores—are small (<1500 Da) molecules with high affinity to iron, secreted by many microorganisms. The iron-free form chelates iron in the environment and the iron–chelator complex is transported back into the producer organism. According to their chemical structure, iron-chelators can be classified into chatecolates, carboxylates, and hydroxamates. Almost all fungal iron chelators are hydroxamates (Renshaw et al. 2002, Winkelmann 2007) except rhizoferrin (carboxylate), produced by the phylum Zygomycetes (Thieken and Winkelmann 1992).

**Figure 2.**
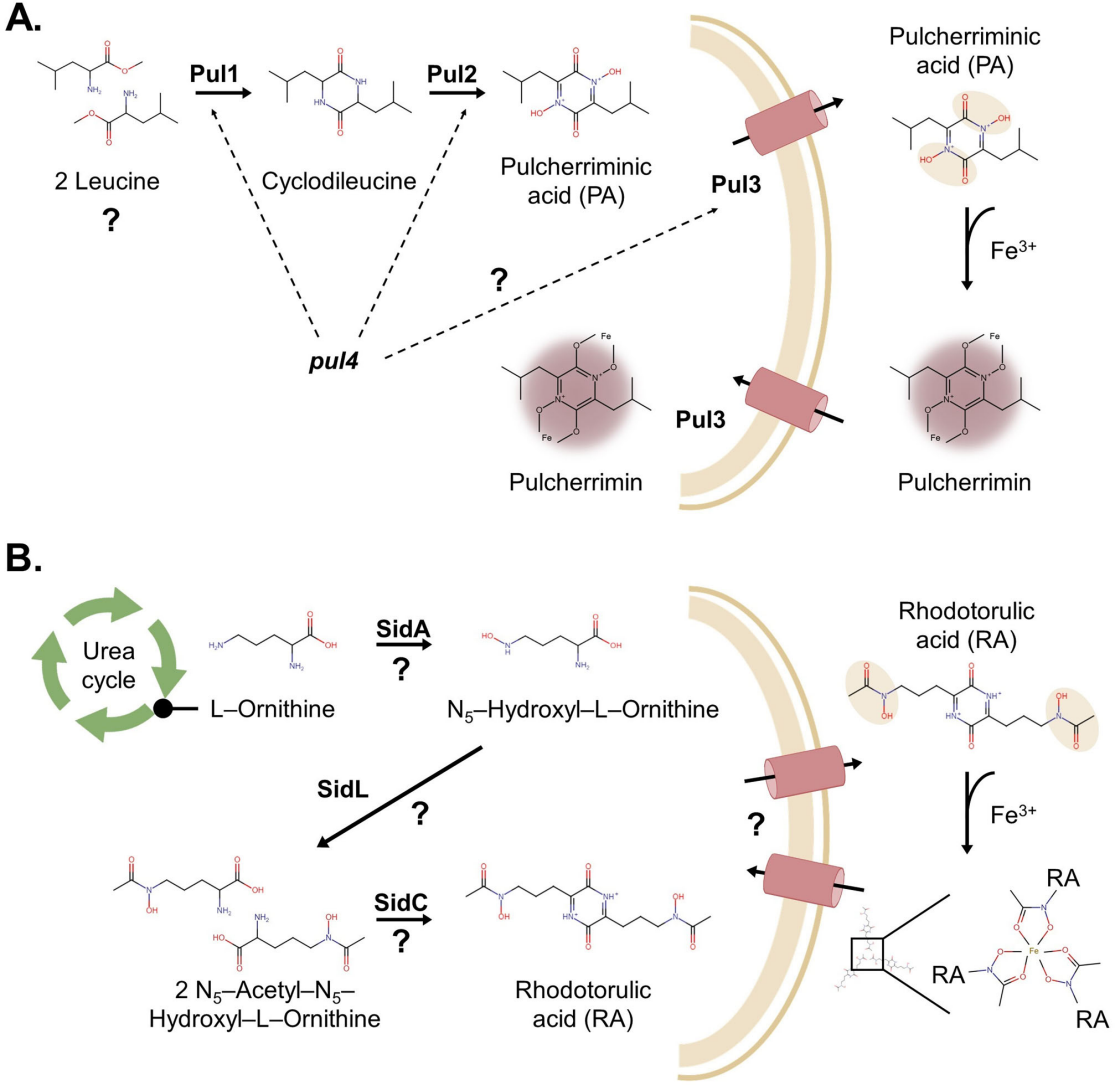
Biosynthesis of the yeast-based iron chelators PA (A) and RA (B). The hydroxamate groups are shaded in beige. The question mark (?) highlights unknown molecular aspects. Pulcherrimin, the iron-bound form of PA, is shaded in maroon as it adopts a characteristic maroon coloration. (A) Biosynthesis of PA: two molecules of leucine—unclear if free or tRNA**-**charged—are cyclized into cyclodileucine by Pul1 (pul1), which is oxidized by Pul2 (pul2) to yield PA. PA is exported by the pul3-encoded transporter Pul3 and imported when bound to iron by the same protein. The iron-bound form is known as pulcherrimin and adopts a characteristic maroon coloration. Pul4 (pul4) positively regulates the expression of pul1 and 2; this gene also affects pul3 expression but is not essential for its expression. (B) Biosynthesis of RA: l-ornithine produced in the urea cycle is oxidized, transacylated, and condensed to form RA. The genes involved are unknown but can be inferred from the genomes of Rhodotorula species based on their homology to the *Aspergillus fumigatus* biosynthesis genes for ferricrocin production, a similar hydroxamate siderophore (Table S1). The import/export mechanism of RA is unknown. When bound to iron, three molecules of RA coordinate their functional groups. The biosynthesis of RA is induced under iron-limiting conditions.

Focussing on yeast, the most studied iron chelators are pulcherriminic acid (PA) (Fig. 2A)—produced mainly by *Metschnikowia* and *Kluyveromyces* species (Ascomycota) (Araujo and Hagler 2011, Krause et al. 2018, Sipiczki 2020, Mažeika et al. 2021); rhodotorulic acid (RA) (Fig. 2B)—produced by *Rhodotorula* species (Basidiomycota) (Atkin et al. 1970); and fusarinine C, also known as fusigen, produced by the yeast-like fungus *Aureobasidium* spp. (Wang et al. 2009a, b, Lu et al. 2019). To the best of our knowledge, fusarinine C has only been tested against marine pathogenic bacteria and not against fungi (Wang et al. 2009a, b), therefore, within the scope of this review, we will focus on PA and RA (Fig. 2), which have been extensively tested for their antifungal activity against both human and plant pathogens, including *Botrytis cinerea, Alternaria alternata, Brettanomyces bruxellensis, Penicillium* spp., *Candida* spp., *Zygosaccharomyces* spp., and *Aspergillus* spp. (Calvente et al. 2001, Sipiczki 2006, Saravanakumar et al. 2008, Oro et al. 2014, Türkel et al. 2014, Parafati et al. 2015, Kántor et al. 2016, Pretscher et al. 2018).

Structurally, PA and RA are cyclic dipeptides (CDPs) with two hydroxamate functional groups each (Kluyver et al. 1953, Atkin and Neilands 1968). The hydroxamates strongly bind positively charged metal ions when deprotonated. In the case of PA, the hydroxamic acid groups are found within the backbone of the structure, while in the case of RA they are found as pendant radicals with 10-atom distance between each other (Fig. 2). The hydroxamates harboured by RA form a triply bridged structure with radicals from other two RA molecules (Boukhalfa et al. 2000).

Mechanistically, both compounds display fungistatic antagonism based on starving competitors for iron: In 2001, Calvente et al. (2001) showed for the first time an association between the presence of RA and a reduction of conidial germination in *B. cinerea *. In 2006, Sipiczki (2006) showed that the antimicrobial activity of PA was iron-dependent, given that iron supplementation reduced growth inhibition of *B. cinerea *. Since then, several researchers have observed an iron-dependent correlation between PA production and antifungal activity (Saravanakumar et al. 2008, Türkel and Ener 2009, Oro et al. 2014, Parafati et al. 2015, Kántor et al. 2016, Pretscher et al. 2018).

Noteworthy, while iron chelators secreted by *Rhodotorula* species have been chemically confirmed to be RA (Calvente et al. 1999, 2001, Sansone et al. 2005), for PA we found that many ascomycetous yeasts, which were reported to display an iron-dependent antagonism, the produced molecule was only assumed to be PA without further chemical characterization (Wang et al. 2009a, Atkin and Neilands 1968, Van Der Walt et al. 1990, Schrettl et al. 2004) (Table S2). It would be worthwhile to characterize the chemical structure of those yeast isolates: some reported isolates have shown different target spectra (Sipiczki 2006, Saravanakumar et al. 2008, Kántor et al. 2016, Pretscher et al. 2018) (Table S2) and given both bacteria (Schalk 2025) and filamentous fungi (Renshaw et al. 2002) are known to produce a diverse variety of iron chelators, novel yeast-based iron chelators with antifungal activity could be identified.

### Biosynthesis and regulation of PA and RA

PA and RA are both CDPs (Fig. 2). CDPs are a broad class of bioactive compounds produced by many organisms with applications in medicine, biocatalysis, and material sciences (Widodo and Billerbeck 2023). CDPs can be synthesized from modified or nonmodified amino acids precursors through two unrelated enzyme families: nonribosomal peptide synthetases (NRPS) or cyclodipeptide synthetases (CDPS). Interestingly, PA and RA represent examples of both biosynthetic strategies: PA is produced by a CDPS and RA, by an NRPS.

Although PA was first discovered in yeast (Kluyver et al. 1953), knowledge of its chemical structure and biosynthesis has been mainly studied in the Gram-positive bacteria *Bacillus subtilis* and *Bacillus licheniformis* (Cryle et al. 2010, Bonnefond et al. 2011, Randazzo et al. 2016): In short, two tRNA-charged leucine molecules are cyclized by the CDPS YymC, encoded by *yvmC*. Subsequently, a cytochrome P450 oxidase encoded by *cypX* oxidizes cyclodileucine to PA, which can bind iron extracellularly to form the maroon pigment known as pulcherrimin (Fig. 2A).

In yeast, Krause et al. (2018) identified a pulcherrimin biosynthesis gene cluster (*pul1*-*4*) in various pigmented yeasts including *Kluyveromyces lactis* and *Metschnikowia pulcherrima;* and verified its direct role in PA production via targeted gene deletion in *K. lactis *. The chemical structure of the iron chelator was later confirmed to be the same as the bacterial molecule (Gore-Lloyd et al. 2019).

The *pul* gene cluster encodes for four genes (*pul1-4*) with no sequence homology to the biosynthesis genes in bacteria (Krause et al. 2018). *Pul1* is hypothesized to encode the CDPS catalysing the cyclization of two leucine molecules to form cyclodileucine (Fig. 2A). It remains unclear whether free leucine or tRNA-charged leucine is used as the precursor. *Pul2* is a predicted P450 cytochrome, hence hypothesized to catalyse the final oxidation that yields PA. *Pul3* encodes for a putative transporter, essential for both secretion of PA and the uptake of pulcherrimin (Krause et al. 2018, Maciá Valero et al. 2025b) (Fig. 2A). Since a *pul4* deletion abolishes PA production and pulcherrimin uptake, Pul4 is likely a transcription factor positively regulating the *pul* gene cluster (Krause et al. 2018) (Fig. 2A).

Besides the role of *pul4*, rather little is known about the regulation of PA biosynthesis by other genetic and environmental factors. However, regulation would be critically important to understand for environmental biocontrol applications. In *M. pulcherrima*, Snf2 was reported to play a role in PA biosynthesis regulation (Gore-Lloyd et al. 2019). However, since Snf2 is a chromatin remodeller and its deletion entailed global changes in transcriptome levels, its role in regulation of pulcherrimin production is most likely rather nonspecific (Liu et al. 2017, Gore-Lloyd et al. 2019). Further, it was shown that externally added arginine and tryptophan upregulated the *pul* cluster transcription (Wang et al. 2022a, Zhang et al. 2022) but the mechanism behind it remains unclear.

Unlike classical siderophores—which are used for iron-scavenging and whose biosynthesis is consequently downregulated when enough iron is present in the environment—, iron does not repress PA production (Sipiczki 2006, Parafati et al. 2015, Kántor et al. 2016, Krause et al. 2018). Rather the opposite, increase of iron concentration in the media results in a darker cellular pigmentation of PA producers (Saravanakumar et al. 2008, Oro et al. 2014, Parafati et al. 2015, Kántor et al. 2016, Pretscher et al. 2018). This colour is formed extracellularly once PA and iron form the complex pulcherrimin (Parafati et al. 2015, Kántor et al. 2016). Subsequently, pulcherrimin is transported back into the cell; therefore, cells containing higher intracellular pulcherrimin concentration show darker pigmentation. Consequently, it has been suggested that the ecological function of PA is iron monopolization rather than scavenging (Krause et al. 2018). Measurements on transcription levels of *pul* genes with different iron concentrations should follow.

The precursors of RA were first identified in *Rhodotorula piliminae* using protonated amino acids (Akers et al. 1972) and they are shared with other fungal hydroxamate siderophores, such as fusarinine C or ferricrocin from *Aspergillus fumigatus* (Fig. 2B); therefore, it is likely that those organisms share a similar biosynthetic pathway. In a nutshell, l-ornithine coming from the urea cycle is oxidized to N_5_-hydroxyl-l-ornithine; subsequently, this product is either transacylated to produce other chelators, or transacetylated to N_5_-acetyl-N_5_-hydroxyl-l-ornithine, like in the case of RA. Ultimately, two molecules of N_5_-acetyl-N_5_-hydroxyl-l-ornithine are likely condensed to form RA.

The enzymes catalysing the oxidation, transacetylation, and condensation of RA in yeast remain unknown. However, the biosynthesis of hydroxamate chelators has been well-characterized in *A. fumigatus* and *Aureobasidium melanogenum* (Schrettl et al. 2007, Lu et al. 2019). Gene *sidA* encodes an l-ornithine N_5_-oxygensase; *sidL* codifies an l-ornithine-N_5_-transacetylase that incorporates an acetyl-coA group to the molecule; and *sidC* is an NRPS responsible for the condensation of two molecules of N_5_-acetyl-N_5_-hydroxyl-l-ornithine to finally give RA (Fig. 2B). Other cluster genes (s*idF, sidD…*) are involved in siderophore derivatization. In *A. fumigatus, sit1* and *sit2* are essential for the transport of coprogen and ferrichrome-type siderophores, but not for RA (Aguiar et al. 2021); we hypothesize that a similar siderophore transporter is responsible for the export and import of RA. For RA, despite its early discovery and the description of its precursors, its biosynthetic pathway has not been confirmed in yeast, nor the genes involved. We performed a BLAST search for homologous proteins to *A. fumigatus* in species from the genus *Rhodotorula* that revealed a potential biosynthetic pathway (Table S1). These hypothetical enzymes should be experimentally confirmed.

In contrast to PA, the production of RA is repressed in the presence of iron (Atkin and Neilands 1968, Atkin et al. 1970, Anke and Diekmann 1972), a characteristic shared with classical iron siderophores. This regulatory system suggests that the biological function of RA is associated with survival rather than competition. Similarly, siderophore production in *A. fumigatus* is inactivated under iron-rich conditions, where gene *sreA*, a GATA transcription factor, partially mediates repression of *SidA* (among other genes related to iron uptake and metabolism) in an iron-dependent manner, based on Northern blot analysis of a *∆sreA* strain (Schrettl et al. 2008). We blasted SreA from *A. fumigatus* and found homologous proteins for the same *Rhodotorula* spp. found above (Table S1). We hypothesize that these species possess a similar regulatory system. It was also shown that addition of glutamic acid, arginine, and l-ornithine increased RA production in same-genus species *R. glutinis, R. rubra*, and *R. mucilaginosa* (Calvente et al. 2001, Andersen et al. 2003).

### Screening for iron chelator-producing yeasts and next steps towards their application

The use of living iron chelator-producing yeast as biocontrol agents against wine spoilage or postharvest fruit pathogens has been suggested in multiple occasions (Saravanakumar et al. 2008, Oro et al. 2014, Türkel et al. 2014, Parafati et al. 2016). Moreover, biocontrol and medical applications have been outlined for siderophores produced by bacteria (Schalk 2025). Furthermore, isolated iron chelators have been suggested as antimicrobials, either to sequester iron or to use them as siderophore–drug conjugate, a strategy known as the Trojan horse approach (Roosenberg et al. 2000, de Carvalho and Fernandes 2014, Dassonville-Klimpt and Sonnet 2020). In fact, some synthetic iron chelators are approved by the Food and Drug agency (FDA) for the oral intake against pathologies related to excessive iron accumulation (Manish et al. 2025), showcasing the potential of these compounds in human health. However, there is a need of novel alternatives due to their short half-life in plasma and toxic side effects (Manish et al. 2025).

To facilitate product development involving living yeast cells, thorough characterization is required: many antifungal isolates do not only show one mode to inhibit fungi, but use many confounding mechanisms, making molecular disentangling of individual traits important for product development. Next to secreted antifungal molecules, other modes such as competition for nutrients and space via biofilm formation or stimulation of immune response in plants have been hypothesized (Sipiczki 2006, Kántor et al. 2016, Spadaro and Droby 2016, Freimoser et al. 2019).

A starting point for disentangling which secreted molecules are contributing to the antifungal phenotype is using assays that can discriminate between the different molecule classes (iron chelators, VOCs, biosurfactants, mycocins, and hydrolytic enzymes). In a previous biomining effort, we used an iron depletion halo assay (Fig. 1) (Prins and Billerbeck 2025) that compared the inhibition capacity of a yeast isolate on iron-supplemented media against its inhibition capacity under iron-limiting conditions; this constituted a straightforward strategy to identify yeast isolates whose inhibitory activity relied on iron monopolization (Maciá Valero et al. 2025b).

In a next step, selectively deleting biosynthetic pathways or transferring them into genetically tractable chassis strains like *Saccharomyces cerevisiae* can then be used to prove that a compound is necessary and sufficient for the antifungal phenotype. The latter strategy has been successfully employed for PA by its heterologous production in *S. cerevisiae* and showing its production necessary and sufficient for inhibitory effects against *Botrytis* conidiation (Freimoser et al. 2023) and *C. auris* (Maciá Valero et al. 2025b). To the best of our knowledge, the heterologous production of RA has not been attempted.

These heterologous systems could also allow for high yield production of iron chelators via metabolic engineering and lead to a better understanding of pathway regulation, e.g. the precise role of *pul4* (Fig. 2A). Deeper knowledge on regulation would allow to better understand and consequently control conditions that negatively regulate PA production in an environmental application.

### VOCs produced by yeast: structure and mechanism of inhibition

VOCs are small carbon-based molecules, often smaller than 300 Da, with a low polarity and a high vapour pressure at ambient conditions (Korpi et al. 2009). VOCs are chemically very diverse, including alcohols, esters, ketones, and benzene derivatives.

Both bacteria and fungi naturally produce VOCs and, rather than producing one single compound, these microorganisms often produce several compounds, referred to as the ‘volatilome’. The microbial volatilome has diverse biological functions: promotion of plant growth, induction of plant resistance mechanisms, quorum sensing, killing of plant parasites, and inhibition of fungal phytopathogens (Naznin et al. 2014, Fincheira et al. 2021, De Clerck et al. 2022, Deng et al. 2022, Kowalska et al. 2022). Within the scope of this review, we will focus on yeasts producing VOCs with antifungal activity (previously reviewed here, Morath et al. 2012, Freimoser et al. 2019, Tilocca et al. 2020, Kowalska et al. 2022, Zhao et al. 2022).

The natural production of VOCs has been reported in many yeast species, including *Aureobasidium pullulans, Candida albicans, Hanseniaspora uvarum, Lachancea thermotolerans, M. pulcherrima, S. cerevisiae*, and *Wickerhamomyces anomalus* (Derengowski et al. 2009, Fialho et al. 2010, Liu et al. 2014, Ruiz-Moyano et al. 2020, Di Francesco et al. 2024), with antifungal activity against phytopathogens and food spoilers such as *Aspergillus carbonarius, A. flavus, B. cinerea*, and *Penicillium* species (Masoud et al. 2005, Fialho et al. 2010, Liu et al. 2014, Parafati et al. 2015, Farbo et al. 2018, Ruiz-Moyano et al. 2020, Tejero et al. 2021, 2022, Di Francesco et al. 2024). Further examples are listed in Table S2.

**Figure 3.**
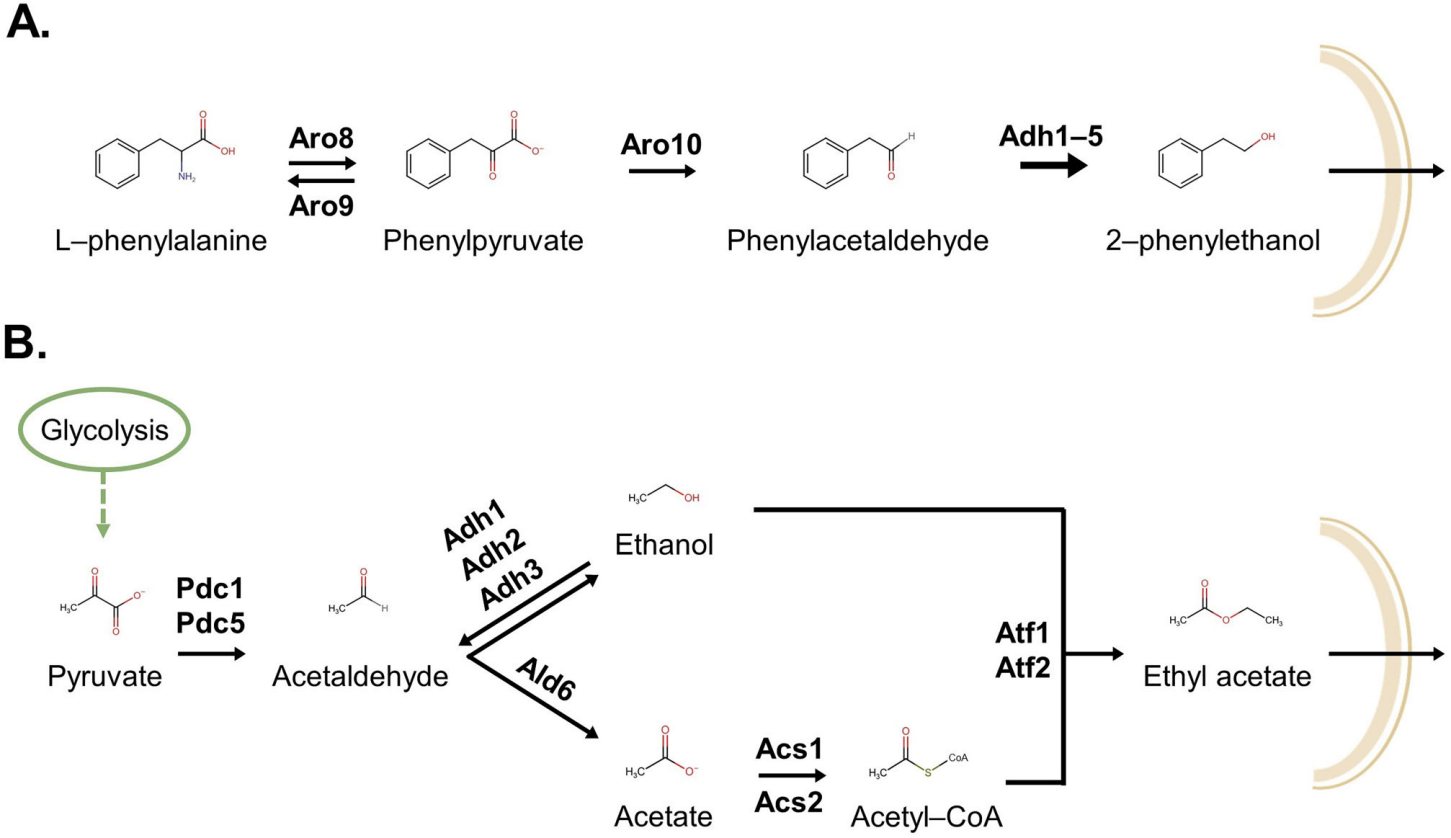
Biosynthesis of the antifungal VOCs 2-phenylethanol (A) and ethyl acetate (B). Both VOCs can freely diffuse through the membrane. (A) Biosynthesis of 2-phenylethanol: l-phenylalanine is transaminated into phenylpyruvate by Aro8 and Aro9; Phenylpyruvate is then decarboxylated by Aro10 to yield phenylacetaldehyde. Finally, this product is reduced to 2-phenylethanol by one of the six alcohol dehydrogenases from *S. cerevisiae*, Adh1, Adh2, Adh3, Adh4, Adh5, or Adh6 (Adh1-6). (B) Biosynthesis of ethyl acetate: Pyruvate gets decarboxylated to yield acetaldehyde by Pdc1, Pdc5, or Pdc6. The resulting acetaldehyde is either converted to ethanol by a reversible alcohol dehydrogenase Adh1, Adh2, or Adh3 or converted into acetate by Ald6, an aldehyde dehydrogenase. Acetate is a substrate for the synthesis of acetyl-CoA by acetyl-coenzyme A synthetases Acs1 and Acs2. For the eventual synthesis of ethyl acetate, acetyl-CoA and ethanol are esterified by alcohol acetyltransferases Atf1 and/or Atf2.

We will illustrate this group of compounds with 2-phenylethanol and ethyl acetate as representative examples (Fig. 3). The compound 2-phenylethanol, also called phenylethyl alcohol, is a primary alcohol with a benzene radical; it has several modes of action including cell membrane disruption, reduction of reactive oxygen species (ROS) and inhibition of biofilm growth (Balbino et al. 2021, Zou et al. 2022, Hof et al. 2023). Ethyl acetate is the ethyl ester formed by ethanol and acetic acid; its mechanism of action against fungal pathogens can rely on inhibition of spore germination, mycelial growth and biofilm formation, cell membrane disruption, and downregulation of metabolism (Wang et al. 2022b, Liu et al. 2022, Masuku et al. 2023).

### Biosynthesis and regulation of 2-phenylethanol and ethyl acetate

In terms of biosynthesis, 2-phenylethanol can be formed *de novo* from prephenate coming from the Shikimate pathway (Yang et al. 2025). However, the most common biosynthesis route is part of the Ehrlich pathway, constituted by three reactions: a transamination, a decarboxylation, and a reduction (Fig. 3A). The knowledge on these reactions and the genes involved is derived from studies in *S. cerevisiae*: first, l-phenylalanine is converted into phenylpyruvate by the two transaminases Aro8 and Aro9 (encoded by *aro8* and *aro9*, respectively) (Fig. 3A) (Iraqui et al. 1998, Karsten et al. 2011, Wang et al. 2017). Then, phenylpyruvate is decarboxylated to phenylacetaldehyde by an enzyme encoded by *aro10* (Vuralhan et al. 2003). Phenylacetaldehyde is eventually reduced to 2-phenylethanol by different alcohol dehydrogenases, Adh1, Adh2, Adh3, Adh4, Adh5, or Adh6, encoded by *adh1, adh2, adh3, adh4, adh5*, or *adh6*, respectively (Larroy et al. 2002, 2003, Dickinson et al. 2003) (Fig. 3A).

The biosynthesis of 2-phenylethanol via the Ehrlich pathway is simplified in Fig. 3(A), it involves more metabolic reactions and an extensively studied regulation system (Dai et al. 2021). In short, the expression of Aro8, catalysing the first transaminase step is constitutively expressed, while Aro9 and Aro10 are upregulated in the presence of aromatic amino acids (l-phenylalanine, tryptophan, or tyrosine) (Iraqui et al. 1998). The expression of the different alcohol dehydrogenases is regulated by different transcription factors and carbon sources. More detailed information can be found here (de Smidt et al. 2008, Gutiérrez-Corona et al. 2023).

For the biosynthesis of ethyl acetate, in *S. cerevisiae*, pyruvate is decarboxylated by the pyruvate decarboxylase Pdc1 or Pdc5 (encoded by *pdc1* or *pdc5*, respectively) to form acetaldehyde (Flikweert et al. 1996, Vuralhan et al. 2003) (Fig. 3B). Then, acetaldehyde can be converted to ethanol (or vice versa) depending on oxygen availability; this reversible reaction is catalysed by Adh1, Adh2, or Adh3, different isoforms of an alcohol dehydrogenase encoded by *adh1, adh2*, and *adh3*, respectively (de Smidt et al. 2012). Another parallel reaction is the decomposition of acetaldehyde into acetate by aldehyde dehydrogenases, mainly cytosolic Ald6 (encoded by *ald6*) in acetate (Saint-Prix et al. 2004). Subsequently, acetate is used as a substrate by acetyl-coenzyme A synthetases Acs1 and Acs2 (*acs1* and *acs2*, respectively) to form acetyl-CoA (van den Berg et al. 1996, Takahashi et al. 2006). The final esterification of acetyl-CoA and ethanol to form ethyl acetate can be catalysed by Atf1 or Atf2, both alcohol acetyltransferases encoded by *atf1* and *atf2*, respectively (Verstrepen et al. 2003) (Fig. 3B).

The regulation of ethyl acetate biosynthesis comprises an extensively studied complex regulatory network. In brief, regulation of the first reaction catalysed by the two pyruvate decarboxylases Pdc1 and Pdc5 is dependent on growth state: in actively fermenting cells, Pdc1 shows high expression levels while Pdc5 expression is hardly detectable. Interestingly, deletion of the *pdc1* gene leads to strongly enhanced Pdc5 expression (Eberhardt et al. 1999). Moreover, both genes have been reported to be upregulated by the transcription factor Pdc2 (Mojzita and Hohmann 2006). Further, Pdc1 expression is reported to be upregulated by Znf1 during glycolytic fermentation (Songdech et al. 2020) and downregulated by transcription factor Ert1 during gluconeogenesis (Gasmi et al. 2014). For the different alcohol dehydrogenases, detailed information on their expression regulation has been reviewed before (de Smidt et al. 2008, Gutiérrez-Corona et al. 2023). For example, Ald6 expression is regulated by different transcription factors, one of them being Ino2, a regulator involved in the cellular response to glucose starvation (Bergenholm et al. 2018). The acetyl-CoA synthetases Acs1 and Acs2 are regulated by different transcription factors depending on external stimuli, such as the carbon source or stress response (van den Berg et al. 1996). Finally, the expression of Atf1 is strongly repressed in the presence of oxygen (Fujiwara et al. 1999), the expression of Atf2 has been less explored.

### Screening for VOC-producing yeasts and next steps towards their application

The production of VOCs in antagonistic yeast is mainly studied for their application as biocontrol agents against plant pathogens in agriculture and for the treatment of postharvest fruits (Table S2).

In analogy to discussed before, a first step in identifying new antifungal VOC-producing yeasts or verifying if an antifungal phenotype is at least partly due to VOCs a simple dual plate assay can be performed: here, one plate contains the target pathogen and the other one contains the organism of interest (Fig. 1); by having these plates facing each other with no physical contact, and sealed with parafilm, volatile compounds can be concluded as responsible for at least part of the inhibition observed (Huang et al. 2011, Di Francesco et al. 2015). This technique allows the researcher to test a positive hit against a range of pathogens; however, high-throughput screening strategies for a large set of yeasts are missing.

The identification of individual VOCs as the single cause for fungal inhibition is challenging, since the microbial volatilome is highly dynamic, dependent on the growth phase, strain, or media composition (Alves et al. 2015, Fairbairn et al. 2017, Tejero Rioseras et al. 2017, Seguinot et al. 2020, Visinoni et al. 2022). For instance, *Candida intermedia* produced more than 20 VOCs when tested against *A. carbonarius* and *Aspergillus ochraceus*, but 2-phenylethanol was found to be the significantly most abundant VOC present (Farbo et al. 2018). However, when tested against *B. cinerea¸* 18 VOCs were identified, from which seven, including 2-nonanone, 1,3,5,7-cyclo-octatetraene but not 2-phenylethanol, successfully inhibited conidia germination (Huang et al. 2011). While 2-phenylethanol did inhibit the mycelial growth of *B. cinerea*, this VOC was not the most potent against the phytopathogenic fungus (Huang et al. 2011). Different strains of *A. pullulans* also showed a different effect on target species, since 2-phenylethanol was the most efficient VOC against *Colletotrichum acutatum* but 3-methyl-1-butanol was the most active compound against *B. cinerea* (Di Francesco et al. 2015, 2020). The observed inhibitory effect when screening isolates is most likely the result of an additive or synergistic interaction among different VOCs. Although individual VOCs can be tested in isolation to confirm their specific roles, the volatilome composition is highly context-dependent. Thus, for practical applications—particularly those involving living yeasts as biocontrol agents—research should rather focus on identifying minimal active concentration of the volatilome, rather than that of a single compound.

Due to the volatile nature of VOCs, the establishment of inhibitory concentrations and the reproduction of results from the laboratory to an open field for agricultural applications are major challenges. As such, the scientific community suggests the use of yeasts producing VOCs for the preventive treatment of air-tight closed postharvest fruits. Here, similar results have been obtained when using VOC-producing yeast against pathogens on agar plates and when using the same yeast against pathogens on fruit. For example, *A. pullulans* partially protected grapes artificially infected with *B. cinerea* conidia (Di Francesco et al. 2020). A similar result was observed when strawberries inoculated with *B. cinerea* were treated with *C. intermedia* in desiccators; treated strawberries showed 29% rotting incidence compared to untreated fruits (Huang et al. 2011). Experiments of biofumigation with VOCs produced by *H. uvarum* to protect tomatoes also showed successful results against *Phytophthora nicotianae* (Liu et al. 2022).

Another valuable avenue towards application of VOCs is enhancing their biosynthesis through metabolic engineering of natural or heterologous hosts, such engineered producers could be used in contained formats in factories or for postharvest control (Jones et al. 2024). Although performed for biotechnological applications other than antifungal development, metabolic engineering has been shown to enhance the production of the previously exemplified VOC 2-phenylethanol. Some common strategies for metabolic engineering used are the increased uptake of the precursor l-phenylalanine or the blockage of either precursor-competing pathways and/or additional oxidation steps of phenylacetaldehyde in the natural producers *S. cerevisiae, Pichia pastoris*, and *Yarrowia lipolytica* (Wang et al. 2018, Gu et al. 2020, Kong et al. 2020, Zhu et al. 2021). Highest yield observed was 6.3 g/l in *S. cerevisiae* after the expression of the metabolic module under different promoter strengths (Wang et al. 2018).

Also the VOC ethyl acetate has been heterologously produced and optimized in *S. cerevisiae*, by expressing four alcohol acetyltransferases from two other yeast species and two plant species reaching yields of 1.69 g/l (Shi et al. 2021). Further work could lead to the development of highly producing or cocktail-producing strains that could be tested for their biocontrol activity against phytopathogens.

Although the chemical synthesis of VOCs like 2-phenylethanol and ethyl acetate has been reported with yields exceeding 98% and 90% substrate conversion, respectively (Kanojiya et al. 2014, Deng et al. 2016, Sasu et al. 2016, Šulgan et al. 2020), biological production may represent a more sustainable and environmentally friendly alternative, as it operates under milder conditions and could utilize renewable bio-based feedstocks.

### Biosurfactants produced by yeast: structure and mechanism of inhibition

Biosurfactants are surface-active molecules with amphiphilic character, with a hydrophilic head bound to a hydrophobic hydrocarbon chain (Amaral et al. 2010). Biosurfactants are produced by many microorganisms, including bacteria, filamentous fungi, and yeast, and they are generally classified as glucolipids, phospholipids, and lipopeptides. Although fungal biosurfactants represent only 19% of the known scientific output, they show the widest chemical diversity, with some of those being exclusively produced by fungi (da Silva et al. 2021). Further, several studies have shown the antifungal activity of yeast biosurfactants (Kulakovskaya et al. 2003, de O Caretta et al. 2022) (Table S2).

While biosurfactant biosynthesis has been studied longer in bacteria than in yeast, exploring yeasts as surfactant producers offers opportunities: several biosurfactant-producing yeasts have GRAS status (generally regarded as safe) and some yeasts produce biosurfactants at much higher concentrations than bacteria. For example, the optimized batch production of sophorolipid in the bacterial producer *Pseudomonas mendocina* yielded concentrations of 3.1 g/l (Paul et al. 2022), while concentrations higher than 200 g/l could be achieved when using the natural producer *Starmerella bombicola* (Gao et al. 2013).

The most studied yeast biosurfactants are glucolipids and they can be classified based on their polar moiety into sophorolipids, mannosylerythritol lipids (MEL), cellobiose lipids, trehalose lipids, and xylolipids. We here focus on the biosynthesis of sophorolipids, MELs and cellobiose lipids, since those have been best characterized in terms of their biosynthesis and fungal antagonism (Table S2). Further reading on every class can be found elsewhere (Jezierska et al. 2018, da Silva et al. 2021).

Noteworthy, biosurfactants have been mostly studied for applications other than antifungal activity, such as emulsifying agents in cosmetic products, biofertilizers, laundry detergents, or food preservatives (Adetunji and Olaniran 2021). However, several biosurfactant-producing yeast isolates have been successfully tested against fungal pathogens (Table S2). While the mechanism of action of biosurfactants is generally associated with cell membrane disruption, they can show different phenotypic effects: changes in mycelial morphology, hyphae formation, increase of ROS levels, and cellular stress, or the inhibition of mycelial growth, biofilm formation, or/and spore germination (Haque et al. 2016, 2019, Chen et al. 2020, Sen et al. 2020, Kumari et al. 2021).

### Biosynthesis of sophorolipids, MELs, and cellobiose lipids

Knowledge on the biosynthesis of sophorolipids is derived from *S. bombicola*, the most widely used yeast for sophorolipid production (Gao et al. 2013). Other yeast species producing this type of biosurfactant are listed in Table S2.

**Figure 4.**
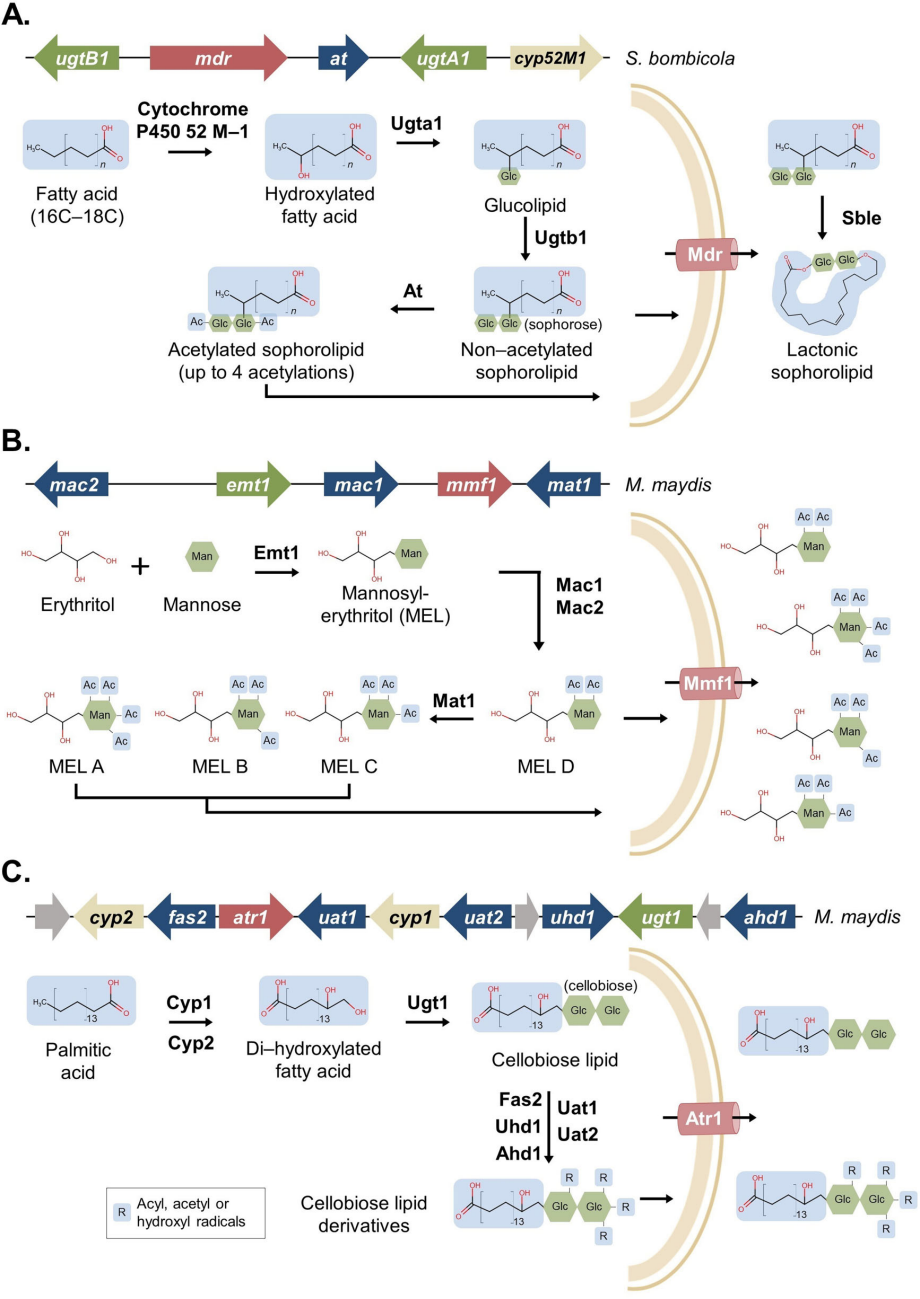
Overview on the biosynthesis of some yeast biosurfactants. Monosaccharides are shaded in green and fatty acids are shaded in blue. (A) Sophorolipid biosynthesis: first, fatty acids are hydroxylated by Cytochrome P450 52-M1. The product gets sequentially glucosylated by Ugta1 and Ugtb1. The resulting sophorolipid can be acetylated by At. Both acetylated (Ac) and nonacetylated sophorolipids are transported to the extracellular via the Mdr transporter. A cell-anchored lactone esterase (Sble) catalyses a transesterification, yielding lactonic sophorolipids. (B) MEL biosynthesis: erythritol gets mannosylated (Man) by Emt1. The resulting MEL product is further acylated (Ac) by Mac1 and Mac2, yielding MEL D (the specific order is unknown). Subsequently, MEL D can be acetylated by Mat1. The resulting biosurfactants are classified into MEL A, B, or C according to the position of acetylation. Mmf1 is hypothesized to be the transporter for MELA, B, C, and D. (C) Cellobiose lipid biosynthesis: palmitic acid gets hydroxylated by Cytochrome P450 1 (cyp1) and the product is further hydroxylated by Cytochrome P450 2 (cyp2). Di-hydroxylated palmitic acid gets glucosylated by Ugt1 and linked to cellobiose. The resulting lipid can either be transported through the Atr1 transporter or be further derivatized via acylation, acetylation or hydroxylation (R) with Fas2, Uat1, Uhd1, or Adh1. The gene cluster from *Mycosarcoma maydis* contains 12 ORFs.

Sophorolipids are constituted of a sophorose dimer (β-1,2-linked glucose) as polar moiety and a hydrophobic tail of 16–18 carbons. For the biosynthesis of sophorolipids, a fatty acid is hydroxylated by a cytochrome P450 52-M1 (encoded by *cyp52M1*) (Van Bogaert et al. 2013) (Fig. 4A). Next, the UDP-glucosyltransferases A1 (encoded by *ugtA1*) attaches one glucose molecule to the free hydroxyl group of the now oxidized fatty acid and UDP-glucosyltransferases B1 (encoded by *ugtB1*) transfers a second glucose molecule to eventually form a sophorose unit linked by an ester bond (Saerens et al. 2011a, b). Subsequently, sophorolipids can be acetylated by a sophorolipid acetyltransferase (encoded by *at*) and, either prior or after acetylation, transported into the extracellular space (Saerens et al. 2011c). Recently, however, Roelants and colleagues observed that acetyltransferase AT was not the only enzyme catalysing sophorolipid acetylation (Roelants et al. 2024). The protein responsible for sophorolipid transport is a putative multidrug resistance protein from the ABC superfamily (*mdr*), but alternative secretion routes might be present. After transport, a cell wall-bound lactone esterase (*sble*) catalyses a transesterification yielding lactonic sophorolipid (Fig. 4A) (Ciesielska et al. 2016).

All sophorolipid biosynthetic genes from *S. bombicola* are located in one gene cluster, except for *sble* (Fig. 4A) (Van Bogaert et al. 2013, Ciesielska et al. 2014). Regulation of this gene cluster is not fully understood, but the protein SbBro1, a homolog of Bro1 from *Y. lipolytica* and *S. cerevisiae*, downregulates the expression of *cyp52M1*, responsible for the first step of sophorolipid biosynthesis (Liu et al. 2020).

MELs are composed of a polar moiety formed by a mannose molecule linked to erythritol and a hydrophobic moiety composed by tail of fatty acids. The most studied yeast species that produce MEL biosurfactants belong to the family Ustilaginaceae (Table S2). Knowledge on the biosynthesis of MEL is derived from the yeast *Mycosarcoma maydis* (Hewald et al. 2006) and fed-batch fermentations have obtained a final yield of 165 g/l in the natural producer *Moesziomyces aphidis* (Rau et al. 2005).

The biosynthesis of MELs starts with the linkage of GDP-activated mannose (mannosylation) to an erythritol molecule, catalysed by a glucosyltransferase (*emt1*) (Fig. 4B) (Hewald et al. 2005). Next, the mannosylerythritol product is acylated by Mac1 (*mac1*) and Mac2 *(mac2*) with short and medium fatty acids. The two enzymes show different region-selectivity and the order of their reaction remains elusive (Hewald et al. 2006). The resulting MELs are known as MEL D. Depending on the yeast species and carbon source, acylated MELs are later acetylated by an acetyltransferase (*mat1*) (Hewald et al. 2006, da Silva et al. 2021). Based on the number of acetylation reactions and the atoms involved in said reactions, the products are classified as MEL A, MEL B, and MEL C (da Silva et al. 2021). After biosynthesis, MELs are likely exported by Mmf1 (*mmf1*), a transmembrane protein from the major facilitator superfamily (Fig. 4B) (Hewald et al. 2006).

The enzymes responsible for the biosynthesis of MELs are also organized in a gene cluster and were first characterized in *M. maydis* (Hewald et al. 2006) (Fig. 4B). Subsequently, homologous MEL clusters have been identified in different basidiomycetes genera (Konishi et al. 2013, Morita et al. 2013, Lorenz et al. 2014, Saika et al. 2014, 2016, Liu et al. 2024). In terms of regulation, it is known that *emt*1 expression is strongly induced under nitrogen starvation conditions (Hewald et al. 2005). Although further studies on genetic expression are needed, several factors are known to improve MEL production, such as hydrophobic carbon sources (vegetable oils; e.g. olive oil or soybean oil), NaNO_3_ as nitrogen source and the presence of Fe^2+^ and Fe^3+^ trace elements in the production media, reviewed here (Valkenburg et al. 2024).

Cellobiose lipids are composed of a polar cellobiose moiety (β-1,4-linked glucose) and a hydrophobic tail of 16 carbons, derived from palmitic acid. Examples of yeast species producing them are listed in Table S2. The characterization of the genes involved in cellobiose lipid biosynthesis first started with *M. maydis* (Teichmann et al. 2007) and was subsequently extended to other yeasts from the family *Ustilaginaceae*, such as *Sporisorium scitamineum* and *Pseudozyma flocculosa* (Teichmann et al. 2011, Oraby et al. 2020). The production of cellobiose lipids in a 1 l bioreactor has reached a yield of 17.6 g/l using *S. scitamineum* (Oraby et al. 2020).

The biosynthesis of cellobiose lipids starts with the hydroxylation of palmitic acid by cytochrome P450 1 and 2 (*cyp1* and *cyp2*) (Teichmann et al. 2007) (Fig. 4C). Cyp1 hydroxylates *de novo* synthesized palmitic acid, then Cyp2 hydroxylates 16-OH-palmitic acid, obtaining 15,16-dihydroxylated palmitic acid (Teichmann et al. 2007). Specific hydroxylations might differ within species. Next, the fatty acid chain is glucosylated with a cellobiose disaccharide by Ugt1 (*ugt1*) (Teichmann et al. 2007). The resulting cellobiose lipid might be subjected to further acylation reactions (*fas2, uat1*, and *uat2*) or hydroxylations (*uhd1* and *adh1*) in different side chains. The final cellobiose lipids are exported by the ABC transporter Atr1 (*atr1*) (Teichmann et al. 2007).

All genes responsible for the biosynthesis of cellobiose lipids in *M. maydis* are organized in a gene cluster constituted by 12 genes (Fig. 4C). In *P. flocculosa*, there are 11 homologous genes (Teichmann et al. 2011). In terms of regulation of cellobiose lipid biosynthesis, the zinc finger transcription factor Rua1 (*rua1*), located upstream *cyp2*, has been shown to activate the gene cluster in *M. maydis* (Teichmann et al. 2010).

### Screening for biosurfactant-producing yeasts and next steps towards their application

Biosurfactants produced by yeast have potential as replacement for synthetic surfactants due to their chemical properties and their ability to prevent microbial growth (Klimek-Szczykutowicz et al. 2024). Biosurfactant-producing yeasts have also been suggested for biocontrol applications in agriculture and for food protection (Haque et al. 2016, 2019, Sen et al. 2020)

As discussed for iron chelators, prior application, a better molecular understanding of surfactant producing yeast is necessary to discriminate between potentially multiple confounding antifungal traits. The first step is using screens that can specifically detect if a yeast in fact produces a biosurfactant. There are several approaches to screen for biosurfactant production (Uyar and Sağlam 2021, Xu et al. 2022, Fernandes et al. 2023) and those could be combined into a two-step workflow: first, a simple HTP-compatible assay for haemolytic activity could be employed using arrayed yeast isolates dotted on a lawn of agar with red blood cells embedded, assaying for a a surrounding halo of lysed red blood cells (Uyar and Sağlam 2021). Second, we suggest the use of an oil displacement assay or a drop collapse assay (Fernandes et al. 2023) (Fig. 1). Both methods require the use of spent media from an isolate rather than cells, so it is not easily scalable. The drop collapse assay has clearer readout, although it requires more material. Nevertheless, as a second step, these assays can be used with only active yeasts obtained in the first step screening to confirm that the secreted compound disrupting the lipid bilayer of red blood cell membranes is a biosurfactant.

Metabolic engineering efforts have already achieved production yields higher than 100 g/l in their natural producer host (Rau et al. 2005, Gao et al. 2013), although the purpose of those studies was not for use as antifungals. To the best of our knowledge, there are no reports on the heterologous production of biosurfactants in a neutral chassis.

### Mycocins produced by yeast: structure and mechanisms of inhibition

Yeast mycocins—historically called yeast killer toxins—comprise a diverse group of proteins with antifungal activity. There are more than 150 yeast species reported to produce mycocins that belong to phyla Ascomycota and Basidiomycota, and include species such as *Saccharomyces, Kluyveromyces, Cyberlindnera, Pichia*, and *Wickerhamomyces*, among others (Klassen et al. 2017). In 2017, Klassen et al. (2017) published an extensive list consolidating the species so far proven to produce mycocins. Some of the most studied mycocins are K1, K2, and K28 produced by *S. cerevisiae*; zymocin, produced by *K. lactis*; and HM-1, by *Cyberlindnera mrakii*.

**Figure 5.**
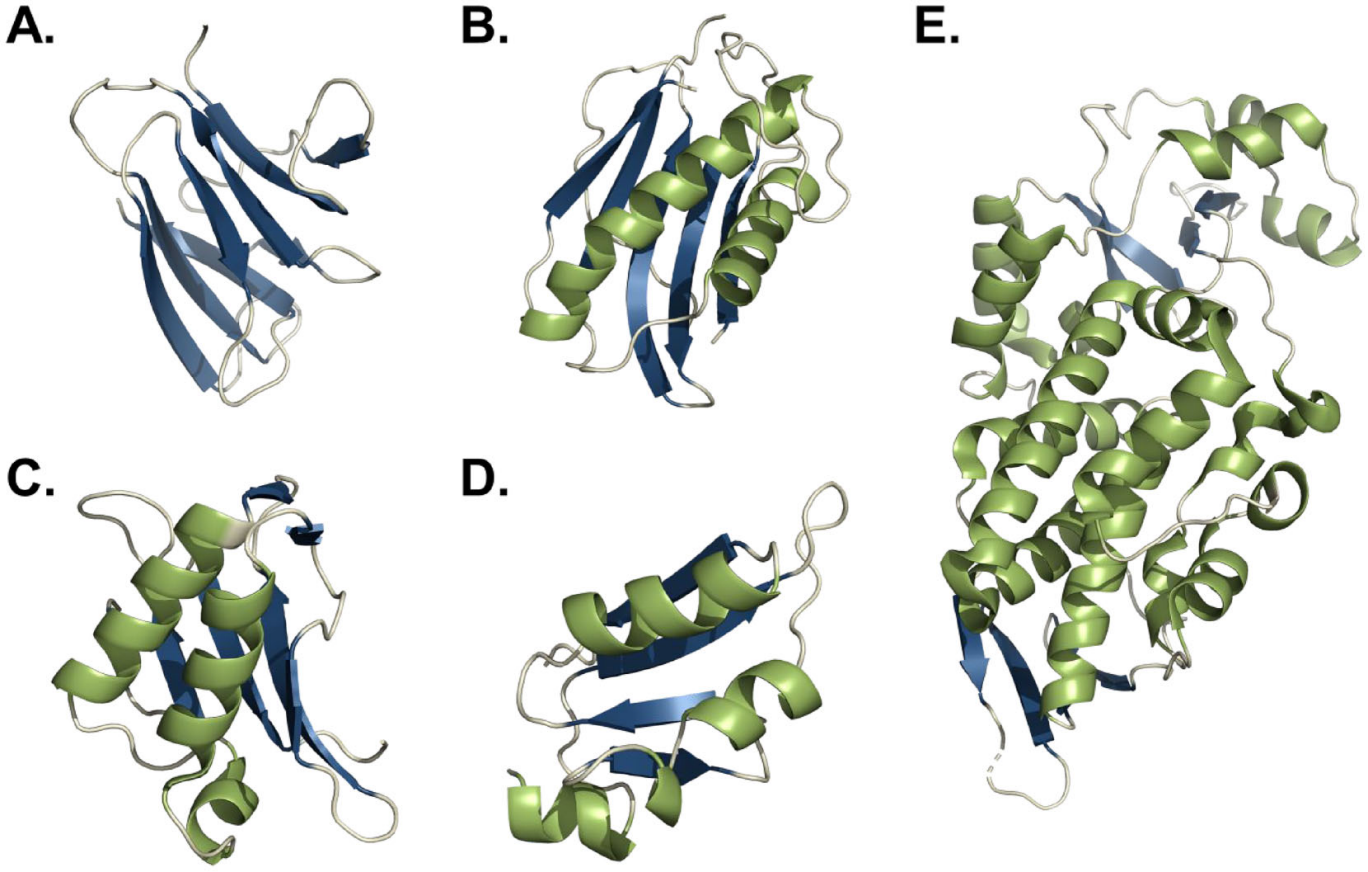
3D structures published for yeast mycocins. Colour scheme indicates secondary structure with α helixes in green, β sheets in dark blue and loops in beige. (A) HM-1 from *C. mrakii* (PDB: 1WKT). (B) SMK from *M. farinosa* (PDB: 1KVD). (C) KP4 from *M. maydis* (PDB: 1KPT). (D) KP6 from *M. maydis* (PDB: 1KP6). and (E) PaT from *M. acaciae* (PDB: 4O87).

Yeast mycocins are diverse in terms of primary sequence and structure. Sizes range from 1.8 (Hodgson et al. 1995) to >150 kDa (Stark and Boyd 1986). Similarly to other secreted proteins, these toxins are translated as pre-pro-toxin precursors, processed during secretion (Klassen et al. 2017). After processing, some mycocins, such as K1, K2, or zymocin, show a heterodimeric composition, with two or more subunits, which were derived from processing of a single precursor (Bostian et al. 1984, Stark and Boyd 1986, Dignard et al. 1991); Others, like HM-1, show only one domain in their mature secreted form (Kimura et al. 1993). Only for five myoccins experimental 3D structures are available: HM-1, SMK (*Millerozyma farinosa*), PaT (*Millerozyma acaciae*), KP4, and KP6 (*M. maydis*) (Gu et al. 1995, Antuch et al. 1996, Kashiwagi et al. 1997, Allen et al. 2013, Chakravarty et al. 2014) (Fig. 5). From those, HM-1 shows an all β-sheet crystalline fold (Fig. 5A), while the remaining four, although dissimilar in sequence, share an α/β-sandwich topology (Fig. 5B–E). More experimental structures could help to understand the structural diversity of mycocins and whether there is a possible classification or correlation with their encoding mechanism or mode of action.

Yeasts that produce mycocins have been reported to show antifungal activity against a variety of food spoilers (Liu and Tsao 2009, Santos et al. 2009, 2010), plant pathogens (Platania et al. 2012, Ferraz et al. 2016, Grzegorczyk et al. 2016), animal pathogens (Guo et al. 2013, Chunming et al. 2018), and human pathogens (Izgü et al. 2007a, b). Some examples are listed in Table S2. Mycocins show diverse modes of actions. For example, both K1 and K2 bind to the fungal cell wall via β-1,6-glucans and kill other yeasts by pore formation (Martinac et al. 1990, Vadasz et al. 2000, Orentaite et al. 2016). K28 also binds to the fungal cell wall and is retrotransported into the cell and to the nucleus of sensitive cells, where it arrests cell proliferation at the G1 phase via DNA synthesis inhibition (Schmitt and Radler 1987, Schmitt et al. 1989, 1996). HM-1 binds to mannoproteins, disrupting the fungal cell wall of sensitive strains (Takasuka et al. 1995, Miyamoto et al. 2012), followed by inhibition of β-1,3-glucan synthase in the plasma membrane, that leads to disruption of cell wall biosynthesis in budding regions and lysis (Yamamoto et al. 1986, Kasaharaa et al. 1994, Komiyama et al. 1996). Zymocin, another mycocin, gets translocated into the cell of sensitive species, where the latter cleaves tRNA, leading to cell cycle arrest of sensitive cells (Butler et al. 1991, Jablonowski et al. 2001, Lu et al. 2005).

### Coding mechanisms and regulation of some well-characterized mycocins

Mycocins are not only diverse in terms of primary sequence and function, but also in terms of their encoding mechanisms. These proteins may be encoded within the nuclear genome or on extrachromosomal elements, such as dsRNA or dsDNA plasmids, with corresponding differences in biosynthesis and regulation.

The best characterized mycocins, K1, K2, and K28, are encoded on dsRNA satellite virus of *S. cerevisiae* (M1, M2, and M28 viruses, respectively) (Hanes et al. 1986, Schmitt and Tipper 1990, Dignard et al. 1991). All three toxins consist of two subunits, α and β, in their mature form (Bostian et al. 1984, Dignard et al. 1991, Schmitt and Tipper 1995) and the same open reading frame also encodes for a self-protection component that confers immunity to the toxin; also called immunity factor (Hanes et al. 1986, Schmitt and Tipper 1990, 1995). In all three cases, a second L–A helper virus is required for replication and encapsidation of both M- and L-viruses, hence for the production of the mycocin as well (Icho and Wickner 1989, Dinman et al. 1991, Fujimura et al. 1992, Schmitt and Tipper 1992). In terms of regulation, most studies indicate that these virus-encoded mycocins are continuously produced and that infection with the virus is sufficient for continued toxin secretion. The possibility that the expression of these mycocins is enhanced during specific growth conditions or by specific additives is unexplored.

Another example with a different encoding mechanism is zymocin. *Kluyveromyces lactis* strains producing zymocin contain two dsDNA virus-like extrachromosomal elements in their cytosol, pGKL1 and pGKL2. Plasmid pGKL1 encodes zymocin and an immunity determinant for the self-protection of producer cells, while pGKL2 is an autonomous ‘helper’ plasmid necessary for replication, maintenance, and expression of pGKL1 (Gunge et al. 1981, Tokunaga et al. 1987). The secreted toxic form is constituted by three subunits (α, β, and γ) and its cytocidal effect relies on the tRNAse activity of the γ subunit (Stark and Boyd 1986). Although the regulation of zymocin production remains underexplored, the increased toxin levels observed upon disruption of *lsm1* and *pab1* in pGKL1 suggest that zymocin is produced continuously in *K. lactis*, consistent with the role of these proteins in mRNA turnover (Vopálenský et al. 2019).

In the case of HM-1, this mycocin is encoded by the gene *hmk*, located in the chromosome of *C. mrakii* (Kasahara et al. 1994). The regulation of its expression and transcription remains to be dissected, similarly to other chromosomally encoded mycocins. It is, however, known that *hmk* gene encodes for a 125-amino acids peptide that, after cellular processing, results in an 88-amino acid mature protein, constituted by one single subunit (Kimura et al. 1993).

Similar to other secreted proteins, mycocins produced by yeast are translated as pre-pro-toxin precursor, which gets processed to the corresponding mature subunits that are sent through the secretion pathway to the extracellular for the execution of their killing activity (Klassen et al. 2017). Although the expression of mycocin-encoding genes seems constitutive, production levels seem highly context-dependent and influenced by environmental factors. For example, some antagonistic yeasts showed an enhanced killer phenotype in the presence of NaCl (Llorente et al. 1997, Al-Qaysi et al. 2017). Ecologically, mycocin production is thought to be favoured in structured environments, where toxins can accumulate locally and reach lethal concentrations, thus providing a selective advantage that outweighs the metabolic cost of production. This advantage is further amplified under nutrient-rich conditions, where the energetic cost of toxin synthesis is less restrictive, whereas under nutrient limitation or in unstructured environments, toxin production provides little to no benefit due to dilution effects and limited contact to sensitive cells (Klassen et al. 2017, Mannazzu et al. 2019).

### Screening for mycocin-producing yeasts and next steps towards their application

Yeast producing mycocins show antifungal activity against a wide range of fungal pathogens and spoilers; consequently, they present substantial potential for different biotechnological applications (Table S2). Most early-discovered and well-studied model mycocins show a narrow pH and temperature profile (Chen et al. 2000, Lebionka et al. 2002, Banjara et al. 2016, Fernández de Ullivarri et al. 2020) and mostly act against *S. cerevisiae*, limiting their application potential (Table S2). However, numerous screens show that killer yeast acting against pathogenic fungi with application-relevant activity profiles can be found in many environments (Baeza et al. 2008, Robledo-Leal et al. 2018, Maciá Valero et al. 2025a).

The first step in screening for mycocin-producing yeast should involve screens that allow to discriminate between yeasts showing antifungal activity that relies on the secretion of a protein and those secreting other compounds with lower molecular weight. We previously discussed a two-step screening for the identification of yeast secreting antifungal proteins within a large set of wild yeast isolates. We first used the general halo assay to rule out isolates that did not show any antifungal activity, then the iron depletion halo assay discussed above to rule out those yeast isolates producing iron chelators (Fig. 1) (Maciá Valero et al. 2025a); We here recommend to perform the iron depletion halo assay to obtain the same level of information with a straightforward readout and run in HTP format (Fig. 1). Yeast isolates that displayed an antifungal iron independent phenotype were then subjected to the next step: here, we used a 3-kDa size exclusion step to discern between those yeasts producing high molecular weight compounds (likely protein) and low-molecular weight compounds (concentrated spent media assay) (Maciá Valero et al. 2025a) (Fig. 1).

Next, additional efforts are required to characterize these candidate isolates producing a novel mycocin. A workflow was proposed by Billerbeck et al. (2024) to identify genes encoding mycocins by combining genomic and proteomic information with genome mutagenesis, this workflow accounts for the low sequence-homology within mycocins. Once an open reading frame (ORF) has been identified, it should be verified via heterologous expression to disentangle the killing phenotype from genotype, which also allows for further study of characteristics from a given mycocin and optimization. The mycocin-encoding genes of those proteins discussed here in detail were heterologously expressed to reconstitute the killing phenotype of the natural producers (Skipper et al. 1984, Dignard et al. 1991, Schmitt 1995, Colussi et al. 2005, Miyamoto et al. 2005).

Once the compound has been cloned and verified to be responsible for the antifungal activity, it can be used in multiple biotechnological applications. For instance, for winemaking, biocontrol, or treatment of vulvovaginal infections in humans, since the optimal conditions for antifungal activity of mycocins are often low temperatures (20°C–25°C) and acidic pH (Chen et al. 2000, Lebionka et al. 2002, Banjara et al. 2016, Fernández de Ullivarri et al. 2020). Some mycocins like HM-1 are functional within a wider range of pH and temperatures, so their use could be expanded to human health. In fact, Selvakumar and colleagues produced monoclonal antibodies able to replicate the fungicidal activity of HM-1 against *Candida* and *Cryptococcus* species (Selvakumar et al. 2006c, a, b, Kabir et al. 2011).

### Hydrolytic enzymes produced by yeast with antifungal activity

The fungal cell is mainly composed of glucans and chitin and most yeasts produce enzymes able to hydrolyze those components for predation, autolysis, morphogenesis, and antagonism.

Although there is no strict differentiation between the previously discussed mycocins and these hydrolytic enzymes—mycocins can have enzymatic activity as well—one distinguishing feature is that these hydrolytic enzymes show high sequence conservation across different taxa and can therefore be easily identified in yeast genomes (Ogrydziak 1993, Roncero and Vázquez de Aldana 2020, Billerbeck et al. 2024). For example, genes *exg1* and *exg2*, encoding β-1,3-exoglucanases, or genes *cts1* and *cts2* encoding endochitinases have been identified in different yeasts (Zhang et al. 2011, Roncero and Vázquez de Aldana 2020). Both Ascomycota and Basidiomycota yeasts produce hydrolytic enzymes and their antifungal activity has been previously reviewed (Table S2) (Freimoser et al. 2019, Roncero and Vázquez de Aldana 2020, Thakur et al. 2023).

The biosynthesis of hydrolytic enzymes follows the conventional path of secreted proteins where an inactive precursor containing an N-terminal signal peptide guides the protein to the cell wall or into the extracellular space, where it exerts its function (Delic et al. 2013). Since one of the main biological functions of hydrolytic enzymes is cell wall morphogenesis during cell division, their synthesis is tightly regulated by the cell cycle machinery. For example, Ace2 is a transcription factor that induces *cts1* expression in *S. cerevisiae* (Dohrmann et al. 1992, King and Butler 1997); its gene *ace2* is only expressed in the G2 phase and Ace2 protein remains in the cytoplasm until late M and early G1 phases, when it starts showing nuclear localization (O’Conalláin et al. 1999). For glucanases, *exg1* expression, for instance, is also regulated by Ace2, but also Mbf and Sbf, other transcription factors expressed in late G1 phase (Iyer et al. 2001).

### Screening for hydrolase-producing yeasts and next steps towards their application

The enzymatic activity of hydrolase-producing yeasts can be used to develop streamlined screening processes. One could test multiple yeast isolates against a target pathogen adding chitin or glucans to agar media for chitinase or exoglucanase activity, respectively (Wu et al. 2018, Gonfa et al. 2023, Kumari et al. 2024). To confirm that the secreted enzyme is, at least partially, responsible for the antifungal activity, inhibitory assays with the spent media of potential candidates concentrated should follow (Maciá Valero et al. 2025b) (Fig. 1). For the screening of peptidase activity, the discrimination is more challenging. Some suggestions are the addition of casein and activity assays with and without protease inhibitors, e.g. EDTA (Ethylenediaminetetraacetic Acid).

Some studies already performed heterologous production of hydrolytic enzymes from yeast. Zhang et al. (2011) expressed *Pgexg1*, encoding an exoglucanase from *Pichia guilliermondii*, in *Escherichia coli* and showed how the recombinant protein successfully controlled the growth of *B. cinerea* in wounded apples. Another study showed that the expression of chitinase-encoding *cts1* and *cts2* genes from *S. cerevisiae* in a tobacco plant reduced by half its susceptibility to *B. cinerea* (Carstens et al. 2003). Those studies disentangle phenotype from genotype and reconstitute the antifungal activity observed in natural producers.

## Conclusions

We here discussed the different antifungal compounds that are known to be produced by yeast and consolidate what is known about their biosynthesis and regulation. While this knowledge hints towards the potential that living yeasts or their molecules could have for biocontrol and human health applications, we highlight the need for better molecular characterization of a given yeast isolate with antifungal activity. Understanding the antifungal activity of a given yeast isolate requires a comprehensive approach that we think can begin with a set of simple screening assays that can inform, which class of molecule contributes to the phenotype. This knowledge can inform searching for relevant gene clusters or single ORFs that encode the active molecule, their functional verification, via heterologous expression in a clean chassis, and/or via deletion in the native host, followed by studying its regulation and mode of action.

We recently suggested a high-throughput compatible screening workflow for antifungal yeasts, that uses simple assays to discriminate between iron chelators and protein producers (Maciá Valero et al. 2025b). In this review, we consolidate additional assays that can be used to cover the other molecule classes, VOCs, biosurfactants, and enzymes. Figure 1 outlines those assays and presents suggested screening workflows for each class of molecule, indicating which approaches are amenable to high-throughput screening in case a user wants to screen a large number of yeast isolates.

In parallel, a deeper understanding of the environmental regulation of antifungal compound biosynthesis is particularly important when deploying living yeasts as biocontrol agents. Knowing whether production is constitutive, environmentally modulated, or specifically triggered by competitors can reveal how yeasts dynamically respond to ecological pressures and inform strategies to optimize compound production and overall efficacy.

To confirm the role of specific compounds in the observed antifungal activities of natural isolates, we highlight studies that use heterologous production in a ‘clean’ host as a viable option. Transfer of a candidate gene cluster or candidate ORF can then be confirmed as necessary and sufficient to transfer (at least part of) the antifungal phenotype. This step could also help elucidate the contribution of individual compounds to the observed effects, as ecological interactions between organisms are often complex, involving multiple antagonistic mechanisms. In Table S2, we highlight examples that even same-species isolates can produce different types of compounds with reported antifungal activity. For example, *S. cerevisiae* can produce VOCs, mycocins and chitinases, *R. glutinis* secretes iron chelators and biosurfactants and *K. lactis* produces both iron siderophores and mycocins. In addition, competition for nutrients and space is an inherent feature of such ecological interactions.

Understanding the specific role of each secreted compound in driving the antagonism could unlock the rational engineering of yeasts optimized either for high secretion titres of single, highly active molecules or for tailored cocktails of compounds. This could be achieved by optimizing the natural host through breeding, random mutagenesis, or engineering, or by leveraging the vast amount of metabolic engineering tools for well-established yeast bioproduction chassis. The resulting crude secretomes or purified molecules of these yeasts hold immediate promise for applications in biocontrol and food preservation. The direct use of optimized yeasts as living agents, however, will remain subject to GMO regulations and the context of the intended application. A recent review offers a forward-looking perspective by proposing an organizational framework that categorizes proof-of-concept studies into three domains factory, farm, and field defined by the extent of microbial interaction with the natural environment and the fundamental challenges and needs for interdisciplinary collaboration that arise from them (Jones et al. 2024).

With increasing insight into the mechanisms underlying antifungal activity, it should become feasible to strategically design and optimize antifungal yeasts to support the next generation of solutions to tackle fungal spoilers or pathogens in agriculture, food, medicine, and industrial biotechnology.

## Supplementary Material

foaf068_Supplemental_File
