# Supplementary material for: Yeast-secreted compounds with antifungal activity—screening, genetic parts, biosynthetic pathways, and regulation
Source: FEMS Yeast Res. 2025 Nov 19;25:foaf068. doi: 10.1093/femsyr/foaf068 (PMC12715866; doi:10.1093/femsyr/foaf068)
Supplement: foaf068_Supplemental_File [file foaf068_supplemental_file.docx]

**Supplementary Material**

**Yeast**–**secreted compounds with antifungal activity – screening, genetic parts, biosynthetic pathways and regulation**

Alicia Maciá Valero^1^, Min Lu^1^ and Sonja Billerbeck^1,2^

^1^*Moleculara Microbiology, Groningen Biomolecular Sciences and Biotechnology Institute, University of Groningen, 9747 AG Groningen, the Netherlands.*

*^2^Department of Bioengineering, Imperial College London, South Kensington Campus, London SW7 2AZ, UK*

^*^Corresponding author: s.billerbeck@imperial.ac.uk

**Supplementary Table 1.** Accession numbers for homologous proteins to SidA, sidL and sidC from Aspergillus fumigatus involved in iron siderophore biosynthesis, found in Rhodotorula species.

|  | ***sidA*** | ***sidL*** | ***sidC*** | ***sreA*** |
| --- | --- | --- | --- | --- |
| ***R. graminis* WP1** | XP_018269057 | XP_018269049 | XP_018268913 | XP_018274591 |
| ***R. mucilaginosa* KR** | KAG0657093 | KAG0657106 | KAG0659201 | KAG0662510 |
| ***R. pacifica* JCM10908** | GAA5982521 | GAA5982542 | GAA5988382 | GAA5980194 |
| ***R. paludigena* BS15** | GJN94077 | GJN94085 | GJN94699 | GJN90815 |
| ***R. sphaerocarpa* JCM8202** | GAA6021216 | GAA6021198 | GAA6026711 | GAA6011674 |
| ***R. taiwanensis* MD1149** | POY74914 | POY74939 | POY72765 | POY71965 |
| ***R. toruloides* NP11** | XP_016270023 | XP_016269525 | XP_016272533 | XP_016271830 |

**Table 2.** Yeast species producing secreted compounds with antifungal activity. The table is divided by class of compound and shows the compound produced, potential application and target species. For iron chelators, some were only described as maroon pigments, therefore marked as ‘Unchar.’(*) highlights articles were the iron chelator was pressumable PA but no chemical characterization was performed.

| **Producer species** | **Compound** | **Potential application** | **Target species** | **Ref.** |
| --- | --- | --- | --- | --- |
| Iron chelators | | | | |
| *Kluyveromyces lactis* | Pulcherriminic acid* | Human health | *Saccharomyces cerevisiae* and *Candidozyma auris* | (Krause *et al.* 2018; Maciá Valero *et al.* 2025) |
| *Metschnikowia isolates* | Unchar. | Human health, food and beverage preservation | *S. cerevisiae, Candida* spp, *C. auris, Brettanomyces bruxellensis, Nakaseomyces glabratus* and Z*ygosaccharomyces rouxii* | (Maciá Valero *et al.* 2025; Maciá Valero, Tabatabaeifar and Billerbeck 2025) |
| *Metschnikowia pulcherrima* | Pulcherriminic acid | Biocontrol | *Alternaria eichhorniae, Botrytis caroliana, Davidiella tassiana, Fusarium* spp, *Gibberella fujikuroi, Metarhizium anisopliae, Monilinia fructicola, Mucor* spp, *Rhizoctonia solani* and *Trichoderma* spp | (Hilber-Bodmer *et al.* 2017; Gore-Lloyd *et al.* 2019) |
| *Metschnikowia pulcherrima* | Pulcherriminic acid* | Biocontrol | *B. cinerea, A. alternata* and *P. expansum* | (Saravanakumar *et al.* 2008) |
| *Metschnikowia pulcherrima* isolates | Pulcherriminic acid* | Food preservation and human health | *Candida* spp*, C. palmioleophila, C. parapsilosis, C. saitoana, Kregervanrija fluxuum, Pichia manshurica, Rhodotorula glutinis* and *Zygosaccharomyces* spp | (Kántor *et al.* 2016) |
| *Metschnikowia pulcherrima* isolates | Unchar. | Biocontrol | *B. cinerea* | (Sipiczki 2006) |
| *Metschnikowia pulcherrima* isolates | Unchar. | Biocontrol, food and beverage preservation | *Aspergillus clavatus, Aureobasidium pullulans, B. cinerea, Starmerella bacillaris zemplinina, Gilbertella persicaria, Mucor* spp and *Rhizopus stolonifer* | (Sipiczki 2006) |
| *Metschnikowia pulcherrima* | Pulcherriminic acid* | Biocontrol | *B. cinerea* | (Pretscher *et al.* 2018) |
| *Metschnikowia pulcherrima* | Pulcherriminic acid* | Biocontrol | *Penicillium* spp and *Geotrichum citri–aurantii* | (Liu *et al.* 2017; Wang *et al.* 2021) |
| *Rhodotorula graminis* | Rhodotorulic acid | Biocontrol | *P. expansum, B. cinerea* and *Cladosporium cladosporoides* | (Calvente *et al.* 2001) |
| *Rhodotorula rubra* | Rhodotorulic acid | Biocontrol | *P. expansum, Botrytis cinerea*  *Cladosporium cladosporoides* | (Calvente *et al.* 2001) |
| *Rhodotorula glutinis* | Rhodotorulic acid | Biocontrol | *P. expansum* | (Calvente, Benuzzi and De Tosetti 1999) |
| *Rhodotorula glutinis* | Rhodotorulic acid | Biocontrol | *B. cinerea* | (Sansone *et al.* 2005) |
| Volatile organic compounds | | | | |
| *Aureobasidium pullulans* | 2–Phenylethanol, ethyl acetate, 3–methyl–1–butanol, isobutyl acetate and more | Biocontrol | *B. cinerea, C. acutatum, Penicillium* spp and *Trichoderma* spp | (Di Francesco *et al.* 2015, 2020, 2024) |
| *Aureobasidium melanogenum* and *Aureobasidium subglaciale* | 2–Phenylethanol, ethyl acetate, 3–methyl–1–butanol, isobutyl acetate and more | Biocontrol | *B. cinerea* | (Di Francesco *et al.* 2020) |
| *Candida albicans* | Farnesol | Biocontrol and human health | *P. expansum* and *Paracoccidioides brasiliensis* | (Derengowski *et al.* 2009; Liu *et al.* 2009) |
| *Candida friedrichii* | 2–Phenylethanol | Food preservation | *A. carbonarius* and *A. ochraceus* | (Farbo *et al.* 2018) |
| *Candida intermedia* | 2–Phenylethanol, 2–nonanone, 1,3,5,7–cyclo–octatetraene, 1–butanol and more | Biocontrol and food preservation | *A. carbonarius, A. ochraceus* and *B. cinerea* | (Huang *et al.* 2011; Farbo *et al.* 2018) |
| *Candida sake* | 2–Phenylethanol and 3–nethylbutyl pentanoate | Biocontrol | *P. expansum, B. cinerea* and *Alternaria* spp | (Arrarte *et al.* 2017) |
| *Cyberlindnera jadinii* | 2–Phenylethanol | Food preservation | *A. carbonarius* and *A. ochraceus* | (Farbo *et al.* 2018) |
| *Hanseniaspora uvarum* | 2–phenylethanol, ethyl acetate and isobutyl acetate | Biocontrol and food preservation | *B. cinerea, Aspergillus flavus, A. ochraceus* and *P. nicotianae* | (Masoud, Poll and Jakobsen 2005; Ruiz-Moyano *et al.* 2020; Tejero *et al.* 2021; Liu *et al.* 2022) |
| *Lachancea thermotolerans* | 2–Phenylethanol | Food preservation | *A. carbonarius* and *A. ochraceus* | (Farbo *et al.* 2018) |
| *Metschnikowia pulcherrima* | Ethyl acetate, isobutyl acetate and 1–butanol | Biocontrol | *B. cinerea* and *Trichoderma* spp | (Parafati *et al.* 2015; Di Francesco *et al.* 2024) |
| *Saccharomyces cerevisiae* | 2–Phenylethanol and ethyl acetate | Biocontrol | *B. cinerea* and *Guignardia citricarpa* | (Fialho *et al.* 2010; Parafati *et al.* 2015) |
| *Pichia klufyveri* | 2–phenylethanol, ethyl acetate and isobutyl acetate | Food preservation | *Aspergillus ochraceus* | (Masoud, Poll and Jakobsen 2005) |
| *Wickerhamomyces anomalus* | 2–phenylethanol, ethyl acetate and isobutyl acetate | Biocontrol and food preservation | *B. cinerea, Aspergillus ochraceus* and *Penicillium roqueforti* | (Masoud, Poll and Jakobsen 2005; Liu *et al.* 2014; Parafati *et al.* 2015) |
| Biosurfactants | | | | |
| *Metschnikowia koreensis* | Sophorolipid | Biocontrol | *Fusarium oxysporum, Rhizoctonia solani* and *Colletotrichum gloeosporioides* | (Kumari *et al.* 2021) |
| *Rhodotorula babjevae* | Sophorolipid | Biocontrol and human health | *Trichophyton mentagrophytes*  *C. gloeosporioides, Fusarium* spp*, Corynespora cassiicola* and *T. rubrum.* | (Sen *et al.* 2017, 2020) |
| *Starmerella bombicola* | Sophorolipid | Biocontrol, food preservation and human health | *C. albicans, Aspergillus* spp, *Fusarium* spp*, Rhizopus* spp, *B. cinerea, Pythium ultimum, Sclerotium rolfsii* and *R. solani* | (Haque *et al.* 2016; Hipólito *et al.* 2020; de O Caretta *et al.* 2022) |
| *Starmerella riodocensis* | Sophorolipid | Human health | *C. albicans* | (Alfian *et al.* 2022) |
| *Wickerhamiella domercqiae* | Sophorolipid | Biocontrol | *Fusarium* spp, *P. ultimum, Pyricularia oryzae, R. solani, Alternaria kikuchiana, Gaeumannomyces graminis, Phytophthora infestans* and *Magnaporthe grisea* | (Chen *et al.* 2020) |
| *Pseudozyma* species | MELs | Biocontrol | *Blumeria graminis, Colletotrichum dematium Glomerella cingulate* and *M. grisea* | (Yoshida *et al.* 2015) |
| *Rhodotorula glutinis* | Rhamnolipid | Biocontrol | *Alternaria alternata* | (Yan *et al.* 2014) |
| *Mycosarcoma maydis* | Cellobiose lipid  (ustilagic acid) | Biocontrol | *B. cinerea* | (Teichmann *et al.* 2007) |
| *Pseudozyma flocculosa* | Cellobiose lipid (flocculosin) | Human health | *C. albicans, N. glabratus, Trichosporon asahii, C. parapsilosis* and *C. neoformans* | (Mimee *et al.* 2005) |
| *Pseudozyma fusiformata* | Cellobiose lipid | Human health | *Solicoccozyma terreus* and *C. neoformans* | (Kulakovskaya, Kulakovskaya and Golubev 2003) |
| Mycocins | | | | |
| *Saccharomyces cerevisiae* | K1 | Beverage preservation and human health | *S. cerevisiae, N. glabratus* | (Bussey, Vernet and Sdicu 1988; Fredericks *et al.* 2021) |
| *Saccharomyces cerevisiae* | K2 | Beverage preservation and human health | *S. cerevisiae, N. glabratus* | (Bussey, Vernet and Sdicu 1988; Fredericks *et al.* 2021) |
| *Saccharomyces cerevisiae* | K28 | Beverage preservation | *S. cerevisiae* | (Schmitt and Tipper 1990) |
| *Saccharomyces cerevisiae* | Klus | Beverage preservation and human health | *S. cerevisiae, Hanseniaspora spp, K. lactis, C. albicans, Candida dubliniensis, Candida kefir* and *Candida tropicalis* | (Rodríguez-Cousiño *et al.* 2011) |
| *Cyberlindnera mrakii* | HM-1 | Beverage and food preservation, human health | *B. bruxellensis, C. albicans, C. auris, C. parapsilosis, C. tropicalis, N. glabratus, Z. rouxii* | (Maciá Valero, Tabatabaeifar and Billerbeck 2025) |
| *Kluyveromyces lactis* | Zymocin | Beverage preservation | *S. cerevisiae* | (Sugisaki *et al.* 1984) |
| *Mycosarcoma maydis* | KP4 | Biocontrol | *Ustilago tritici* and *M. maydis* | (Gu *et al.* 1995; Quijano *et al.* 2016) |
| *Mycosarcoma maydis* | KP6 | Beverage preservation | *B. bruxellensis* | (Santos *et al.* 2010) |
| *Millerozyma acacia* | PaT | Beverage preservation | *S. cerevisiae* | (Chakravarty *et al.* 2014) |
| *Pichia membranifaciens* | PMKT2 | Beverage preservation | *B. bruxellensis* | (Santos *et al.* 2009) |
| *Tetrapisispora phaffii* | KpKt | Beverage and food preservation | *B. bruxellensis, B. californica, L. thermotolerans, H. uvarum, M. pulcherrima, S. cerevisiae, S. bacillaris, S. paradoxus* and *Z. bailii* | (Carboni *et al.* 2020) |
| Hydrolytic enzymes | | | | |
| *Meyerozyma guilliermondii* | Glucanase | Biocontrol | *B. cinerea* | (Zhang *et al.* 2011) |
| *Wickerhamomyces anomalus* | Glucanase | Biocontrol | *B. cinerea* | (Parafati *et al.* 2017) |
| *Saccharomyces cerevisiae* | Chitinase | Biocontrol | *B. cinerea* and *C. acutatum* | (Carstens, Vivier and Pretorius 2003; Lopes *et al.* 2015) |
| *Aureobasidium pullulans* | Alkaline serine protease | Biocontrol | *A. alternata, B. cinerea and P. expansum* | (Banani *et al.* 2014) |

**References**

Alfian AR, Watchaputi K, Sooklim C *et al.* Production of new antimicrobial palm oil-derived sophorolipids by the yeast Starmerella riodocensis sp. nov. against Candida albicans hyphal and biofilm formation. *Microb Cell Fact* 2022;**21**:163.

Arrarte E, Garmendia G, Rossini C *et al.* Volatile organic compounds produced by Antarctic strains of Candida sake play a role in the control of postharvest pathogens of apples. *Biol Control* 2017;**109**:14–20.

Banani H, Spadaro D, Zhang D *et al.* Biocontrol activity of an alkaline serine protease from Aureobasidium pullulans expressed in Pichia pastoris against four postharvest pathogens on apple. *Int J Food Microbiol* 2014;**182**–**183**:1–8.

Bussey H, Vernet T, Sdicu AM. Mutual antagonism among killer yeasts: competition between K1 and K2 killers and a novel cDNA-based K1-K2 killer strain of Saccharomyces cerevisiae. *Can J Microbiol* 1988;**34**:38–44.

Calvente V, Benuzzi D, De Tosetti MIS. Antagonistic action of siderophores from Rhodotorula glutinis upon the postharvest pathogen Penicillium expansum. *Int Biodeterior Biodegrad* 1999;**43**:167–72.

Calvente V, De Orellano ME, Sansone G *et al.* Effect of nitrogen source and pH on siderophore production by Rhodotorula strains and their application to biocontrol of phytopathogenic moulds. *J Ind Microbiol Biotechnol* 2001;**26**:226–9.

Carboni G, Fancello F, Zara G *et al.* Production of a lyophilized ready-to-use yeast killer toxin with possible applications in the wine and food industries. *Int J Food Microbiol* 2020;**335**:108883.

Carstens M, Vivier MA, Pretorius IS. The Saccharomyces cerevisiae chitinase, encoded by the CTS1-2 gene, confers antifungal activity against Botrytis cinerea to transgenic tobacco. *Transgenic Res* 2003;**12**:497–508.

Chakravarty AK, Smith P, Jalan R *et al.* Structure, Mechanism, and Specificity of a Eukaryal tRNA Restriction Enzyme Involved in Self-Nonself Discrimination. *Cell Rep* 2014;**7**:339–47.

Chen J, Liu X, Fu S *et al.* Effects of sophorolipids on fungal and oomycete pathogens in relation to pH solubility. *J Appl Microbiol* 2020;**128**:1754–63.

Derengowski LS, De-Souza-Silva C, Braz S V *et al.* Antimicrobial effect of farnesol, a Candida albicans quorum sensing molecule, on Paracoccidioides brasiliensis growth and morphogenesis. *Ann Clin Microbiol Antimicrob* 2009;**8**:13.

Farbo MG, Urgeghe PP, Fiori S *et al.* Effect of yeast volatile organic compounds on ochratoxin A-producing Aspergillus carbonarius and A. ochraceus. *Int J Food Microbiol* 2018;**284**:1–10.

Fialho MB, Toffano L, Pedroso MP *et al.* Volatile organic compounds produced by Saccharomyces cerevisiae inhibit the in vitro development of Guignardia citricarpa, the causal agent of citrus black spot. *World J Microbiol Biotechnol* 2010;**26**:925–32.

Di Francesco A, Moret E, Cignola R *et al.* Yeasts volatile organic compounds (VOCs) as potential growth enhancers and molds biocontrol agents of mushrooms mycelia. *Fungal Biol* 2024;**128**:1859–67.

Di Francesco A, Ugolini L, Lazzeri L *et al.* Production of volatile organic compounds by Aureobasidium pullulans as a potential mechanism of action against postharvest fruit pathogens. *Biol Control* 2015;**81**:8–14.

Di Francesco A, Zajc J, Gunde-Cimerman N *et al.* Bioactivity of volatile organic compounds by Aureobasidium species against gray mold of tomato and table grape. *World J Microbiol Biotechnol* 2020;**36**:1–11.

Fredericks LR, Lee MD, Eckert HR *et al.* Vaginal isolates of candida glabrata are uniquely susceptible to ionophoric killer toxins produced by saccharomyces cerevisiae. *Antimicrob Agents Chemother* 2021;**65**, DOI: 10.1128/AAC.02450-20.

Gore-Lloyd D, Sumann I, Brachmann AO *et al.* Snf2 controls pulcherriminic acid biosynthesis and antifungal activity of the biocontrol yeast Metschnikowia pulcherrima. *Mol Microbiol* 2019;**112**:317–32.

Gu F, Khimani A, Rane SG *et al.* Structure and function of a virally encoded fungal toxin from Ustilago maydis: a fungal and mammalian Ca2+ channel inhibitor. *Structure* 1995;**3**:805–14.

Haque F, Alfatah M, Ganesan K *et al.* Inhibitory Effect of Sophorolipid on Candida albicans Biofilm Formation and Hyphal Growth. *Sci Rep* 2016;**6**:23575.

Hilber-Bodmer M, Schmid M, Ahrens CH *et al.* Competition assays and physiological experiments of soil and phyllosphere yeasts identify Candida subhashii as a novel antagonist of filamentous fungi. *BMC Microbiol* 2017;**17**:1–15.

Hipólito A, Alves da Silva RA, de Oliveira Caretta T *et al.* Evaluation of the antifungal activity of sophorolipids from Starmerella bombicola against food spoilage fungi. *Biocatal Agric Biotechnol* 2020;**29**:101797.

Huang R, Li GQ, Zhang J *et al.* Control of postharvest Botrytis fruit rot of strawberry by volatile organic compounds of Candida intermedia. *Phytopathology* 2011;**101**:859–69.

Kántor A, Hutková J, Petrová J *et al.* Antimicrobial activity of pulcherrimin pigment produced by Metschnikowia pulcherrima against various yeast species. *J Microbiol Biotechnol Food Sci* 2016;**05**:282–5.

Krause DJ, Kominek J, Opulente DA *et al.* Functional and evolutionary characterization of a secondary metabolite gene cluster in budding yeasts. *Proc Natl Acad Sci U S A* 2018;**115**:11030–5.

Kulakovskaya T V., Kulakovskaya E V., Golubev WI. ATP leakage from yeast cells treated by extracellular glycolipids of Pseudozyma fusiformata. *FEMS Yeast Res* 2003;**3**:401–4.

Kumari A, Kumari S, Prasad GS *et al.* Production of Sophorolipid Biosurfactant by Insect Derived Novel Yeast Metschnikowia churdharensis f.a., sp. nov., and Its Antifungal Activity Against Plant and Human Pathogens. *Front Microbiol* 2021;**12**:678668.

Liu P, Cheng Y, Yang M *et al.* Mechanisms of action for 2-phenylethanol isolated from Kloeckera apiculata in control of Penicillium molds of citrus fruits. *BMC Microbiol* 2014;**14**:12–8.

Liu P, Deng B, Long CA *et al.* Effect of farnesol on morphogenesis in the fungal pathogen Penicillium expansum. *Ann Microbiol* 2009;**59**:33–8.

Liu Y, Wang W, Zhou Y *et al.* Isolation, identification and in vitro screening of Chongqing orangery yeasts for the biocontrol of Penicillium digitatum on citrus fruit. *Biol Control* 2017;**110**:18–24.

Liu Z, Tian J, Yan H *et al.* Ethyl acetate produced by Hanseniaspora uvarum is a potential biocontrol agent against tomato fruit rot caused by Phytophthora nicotianae. *Front Microbiol* 2022;**13**, DOI: 10.3389/fmicb.2022.978920.

Lopes MR, Klein MN, Ferraz LP *et al.* Saccharomyces cerevisiae: A novel and efficient biological control agent for Colletotrichum acutatum during pre-harvest. *Microbiol Res* 2015;**175**:93–9.

Maciá Valero A, Tabatabaeifar F, Billerbeck S. Screening a 681-membered yeast collection for the secretion of proteins with antifungal activity. *N Biotechnol* 2025;**86**:55–72.

Maciá Valero A, van Wageningen JJ, Foster AJ *et al.* Pulcherriminic acid Biosynthesis and Transport: Insights from a heterologous system in Saccharomyces cerevisiae . *FEMS Yeast Res* 2025;**2018**, DOI: 10.1093/femsyr/foaf039.

Masoud W, Poll L, Jakobsen M. Influence of volatile compounds produced by yeasts predominant during processing of Coffea arabica in East Africa on growth and ochratoxin A (OTA) production by Aspergillus ochraceus. *Yeast* 2005;**22**:1133–42.

Mimee B, Labbé C, Pelletier R *et al.* Antifungal activity of flocculosin, a novel glycolipid isolated from Pseudozyma flocculosa. *Antimicrob Agents Chemother* 2005;**49**:1597–9.

de O Caretta T, I Silveira VA, Andrade G *et al.* Antimicrobial activity of sophorolipids produced by Starmerella bombicola against phytopathogens from cherry tomato. *J Sci Food Agric* 2022;**102**:1245–54.

Parafati L, Cirvilleri G, Restuccia C *et al.* Potential Role of Exoglucanase Genes (WaEXG1 and WaEXG2) in the Biocontrol Activity of Wickerhamomyces anomalus. *Microb Ecol* 2017;**73**:876–84.

Parafati L, Vitale A, Restuccia C *et al.* Biocontrol ability and action mechanism of food-isolated yeast strains against Botrytis cinerea causing post-harvest bunch rot of table grape. *Food Microbiol* 2015;**47**:85–92.

Pretscher J, Fischkal T, Branscheidt S *et al.* Yeasts from different habitats and their potential as biocontrol agents. *Fermentation* 2018;**4**, DOI: 10.3390/fermentation4020031.

Quijano CD, Wichmann F, Schlaich T *et al.* KP4 to control Ustilago tritici in wheat: Enhanced greenhouse resistance to loose smut and changes in transcript abundance of pathogen related genes in infected KP4 plants. *Biotechnol Reports* 2016;**11**:90–8.

Rodríguez-Cousiño N, Maqueda M, Ambrona J *et al.* A new wine Saccharomyces cerevisiae killer toxin (Klus), encoded by a double-stranded RNA virus, with broad antifungal activity is evolutionarily related to a chromosomal host gene. *Appl Environ Microbiol* 2011;**77**:1822–32.

Ruiz-Moyano S, Hernández A, Galvan AI *et al.* Selection and application of antifungal VOCs-producing yeasts as biocontrol agents of grey mould in fruits. *Food Microbiol* 2020;**92**:103556.

Sansone G, Rezza I, Calvente V *et al.* Control of Botrytis cinerea strains resistant to iprodione in apple with rhodotorulic acid and yeasts. *Postharvest Biol Technol* 2005;**35**:245–51.

Santos A, Navascués E, Bravo E *et al.* Ustilago maydis killer toxin as a new tool for the biocontrol of the wine spoilage yeast Brettanomyces bruxellensis. *Int J Food Microbiol* 2010;**145**:147–54.

Santos A, San Mauro M, Bravo E *et al.* PMKT2, a new killer toxin from Pichia membranifaciens, and its promising biotechnological properties for control of the spoilage yeast Brettanomyces bruxellensis. *Microbiology* 2009;**155**:624–34.

Saravanakumar D, Ciavorella A, Spadaro D *et al.* Metschnikowia pulcherrima strain MACH1 outcompetes Botrytis cinerea, Alternaria alternata and Penicillium expansum in apples through iron depletion. *Postharvest Biol Technol* 2008;**49**:121–8.

Schmitt MJ, Tipper DJ. K28, a unique double-stranded RNA killer virus of Saccharomyces cerevisiae. *Mol Cell Biol* 1990;**10**:4807–15.

Sen S, Borah SN, Bora A *et al.* Production, characterization, and antifungal activity of a biosurfactant produced by Rhodotorula babjevae YS3. *Microb Cell Fact* 2017;**16**:95.

Sen S, Borah SN, Kandimalla R *et al.* Sophorolipid Biosurfactant Can Control Cutaneous Dermatophytosis Caused by Trichophyton mentagrophytes. *Front Microbiol* 2020;**11**:329.

Sipiczki M. Metschnikowia strains isolated from botrytized grapes antagonize fungal and bacterial growth by iron depletion. *Appl Environ Microbiol* 2006;**72**:6716–24.

Sugisaki Y, Gunge N, Sakaguchi K *et al.* Characterization of a novel killer toxin encoded by a double-stranded linear DNA plasmid of Kluyveromyces lactis. *Eur J Biochem* 1984;**141**:241–5.

Teichmann B, Linne U, Hewald S *et al.* A biosynthetic gene cluster for a secreted cellobiose lipid with antifungal activity from Ustilago maydis. *Mol Microbiol* 2007;**66**:525–33.

Tejero P, Martín A, Rodríguez A *et al.* In vitro biological control of aspergillus flavus by hanseniaspora opuntiae l479 and hanseniaspora uvarum l793, producers of antifungal volatile organic compounds. *Toxins (Basel)* 2021;**13**, DOI: 10.3390/toxins13090663.

Wang S, Zhang H, Ruan C *et al.* Metschnikowia citriensis FL01 antagonize Geotrichum citri-aurantii in citrus fruit through key action of iron depletion. *Int J Food Microbiol* 2021;**357**:109384.

Yan F, Xu S, Chen Y *et al.* Effect of rhamnolipids on Rhodotorula glutinis biocontrol of Alternaria alternata infection in cherry tomato fruit. *Postharvest Biol Technol* 2014;**97**:32–5.

Yoshida S, Koitabashi M, Nakamura J *et al.* Effects of biosurfactants, mannosylerythritol lipids, on the hydrophobicity of solid surfaces and infection behaviours of plant pathogenic fungi. *J Appl Microbiol* 2015;**119**:215–24.

Zhang D, Spadaro D, Valente S *et al.* Cloning, characterization and expression of an exo-1,3-β-glucanase gene from the antagonistic yeast, Pichia guilliermondii strain M8 against grey mold on apples. *Biol Control* 2011;**59**:284–93.
